# Single-cell DNA methylome and 3D genome atlas of human subcutaneous adipose tissue

**DOI:** 10.1038/s41588-025-02300-4

**Published:** 2025-08-20

**Authors:** Zeyuan Johnson Chen, Sankha Subhra Das, Asha Kar, Seung Hyuk T. Lee, Kevin D. Abuhanna, Marcus Alvarez, Mihir G. Sukhatme, Zitian Wang, Kyla Z. Gelev, Matthew G. Heffel, Yi Zhang, Oren Avram, Elior Rahmani, Sriram Sankararaman, Markku Laakso, Sini Heinonen, Hilkka Peltoniemi, Eran Halperin, Kirsi H. Pietiläinen, Chongyuan Luo, Päivi Pajukanta

**Affiliations:** 1 Department of Computer Science, University of California, Los Angeles, CA USA; 2 Department of Computational Medicine, University of California, Los Angeles, CA USA; 3 Department of Human Genetics, David Geffen School of Medicine at UCLA, University of California, Los Angeles, CA USA; 4 Bioinformatics Interdepartmental Program, University of California, Los Angeles, CA USA; 5 Department of Anesthesiology and Perioperative Medicine, University of California, Los Angeles, CA USA; 6 Department of Medicine, University of Eastern Finland and Kuopio University Hospital, Kuopio, Finland; 7 Obesity Research Unit, Research Program for Clinical and Molecular Metabolism, Faculty of Medicine, University of Helsinki, Helsinki, Finland; 8 Eira Hospital, Helsinki, Finland; 9 HealthyWeightHub, Endocrinology, Abdominal Center, Helsinki University Central Hospital and University of Helsinki, Helsinki, Finland; 10 Institute for Precision Health, David Geffen School of Medicine at UCLA, University of California, Los Angeles, CA USA

**Keywords:** Epigenomics, Sequencing, Obesity, Epigenetics

## Abstract

The cell-type-level epigenomic landscape of human subcutaneous adipose tissue (SAT) is not well characterized. Here, we elucidate the epigenomic landscape across SAT cell types using snm3C-seq. We find that SAT CG methylation (mCG) displays pronounced hypermethylation in myeloid cells and hypomethylation in adipocytes and adipose stem and progenitor cells, driving nearly half of the 705,063 differentially methylated regions (DMRs). Moreover, *TET1* and *DNMT3A* are identified as plausible regulators of the cell-type-level mCG profiles. Both global mCG profiles and chromosomal compartmentalization reflect SAT cell-type lineage. Notably, adipocytes display more short-range chromosomal interactions, forming complex local 3D genomic structures that regulate transcriptional functions, including adipogenesis. Furthermore, adipocyte DMRs and A compartments are enriched for abdominal obesity genome-wide association study (GWAS) variants and polygenic risk, while myeloid A compartments are enriched for inflammation. Together, we characterize the SAT single-cell-level epigenomic landscape and link GWAS variants and partitioned polygenic risk of abdominal obesity and inflammation to the SAT epigenome.

## Abstract (web summary)

This multiomic study, including single-nucleus DNA methylation and chromatin conformation matched with single-nuclei RNA sequencing, provides insights into the epigenomic landscape of human subcutaneous adipose tissue.

## Main

The global prevalence of abdominal obesity, defined as an excessive accumulation of adipose tissue in the abdominal region, has been increasing at an alarming rate over the past few decades^1,2^. Abdominal obesity is a known predictor of all-cause mortality, probably because of its increased risk of cardiometabolic disease (CMD) and other adverse pathological conditions^3^. The risks related to abdominal obesity are thought to be partly mediated through obesity-induced low-grade inflammation at the tissue and cell-type level^4^. This has stimulated research interest in investigating the molecular origin of abdominal obesity and related co-morbidities by focusing on SAT, the key fat depot in expanding and buffering against obesity.

SAT is highly heterogeneous and comprises an array of cell types^5^. Single-nucleus RNA sequencing (snRNA-seq) enables the discovery of cell-type-level gene expression patterns in SAT^6,7^. However, this modality is limited to gene expression, even though SAT function is also influenced by epigenomic processes, such as cytosine DNA methylation at CpG sites (mCG)^8^, and chromatin conformation^9^. Previous studies in other tissues have shown that cell-type-level dynamic mCG in gene regulatory regions and gene bodies affect the expression of genes^10^. Furthermore, gene regulatory mechanisms need proper chromatin conformation, which is organized into compartments, domains and loops^11^. However, the cell-type-level epigenomic landscape underlying the extensive heterogeneity in SAT is poorly understood in humans, which also hinders genetic risk assessment of abdominal obesity, the functional basis of which probably includes specific cell-type-level epigenomic sites.

Single-nucleus methyl-3C sequencing (snm3C-seq) has emerged as a powerful and innovative platform to study DNA methylation and chromatin conformation at cell-type resolution^12^. Recent studies identified cell-type-level epigenomic signatures in various complex tissues in humans, such as oocytes^13^, prefrontal^12^ and frontal cortex^14,15^, and other diverse brain regions^10^. Using a similar approach, previous studies have also comprehensively assessed the epigenomes of mouse brain cell types^16–18^. However, cell-type-level epigenomic signatures in the human key fat depot SAT have been poorly studied. To address this important biomedical knowledge gap, we determined cell-type-level DNA methylation, chromatin conformation and gene expression signatures in SAT, assessed the involvement of methylation pathway genes in SAT cell-type-level dynamic methylation patterns, identified cell-type-level hypomethylated region-associated transcription factor (TF) binding motifs and investigated the contribution of variants in SAT cell-type-level epigenomic sites to polygenic risk of abdominal obesity and a related inflammatory marker, blood C-reactive protein (CRP).

## Results

### Multimodal profiling of human SAT cell types

**Fig. 1:**
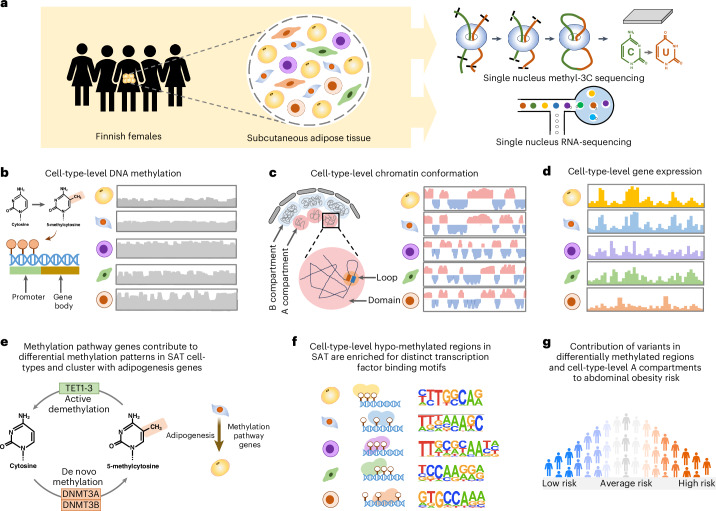
Schematic overview of the study design using snm3C-seq and snRNA-seq to profile cell-type-level DNA methylation, chromatin conformation and gene expression in human SAT and partition the genetic risk of abdominal obesity. **a**, Illustration of snm3C-seq and snRNA-seq on nuclei isolated from SAT biopsies from Finnish females. **b**–**g**, Comprehensive analyses of DNA methylation, chromatin conformation and gene expression profiles across SAT cell types to identify cell-type-level differences in DNA methylation patterns (**b**) and chromatin conformation dynamics (**c**). Subsequently, we used the cell-type-level SAT expression data (**d**) to determine whether methylation pathway genes contribute to the observed differences in methylation patterns in SAT cell types and longitudinally cluster with adipogenesis pathway genes (**e**), identify cell-type-level TF binding motifs associated with hypomethylated regions in SAT cell types (**f**) and test the contribution of variants in cell-type-level DMRs and A and B compartments to the genetic risk of abdominal obesity (**g**).

**Fig. 2:**
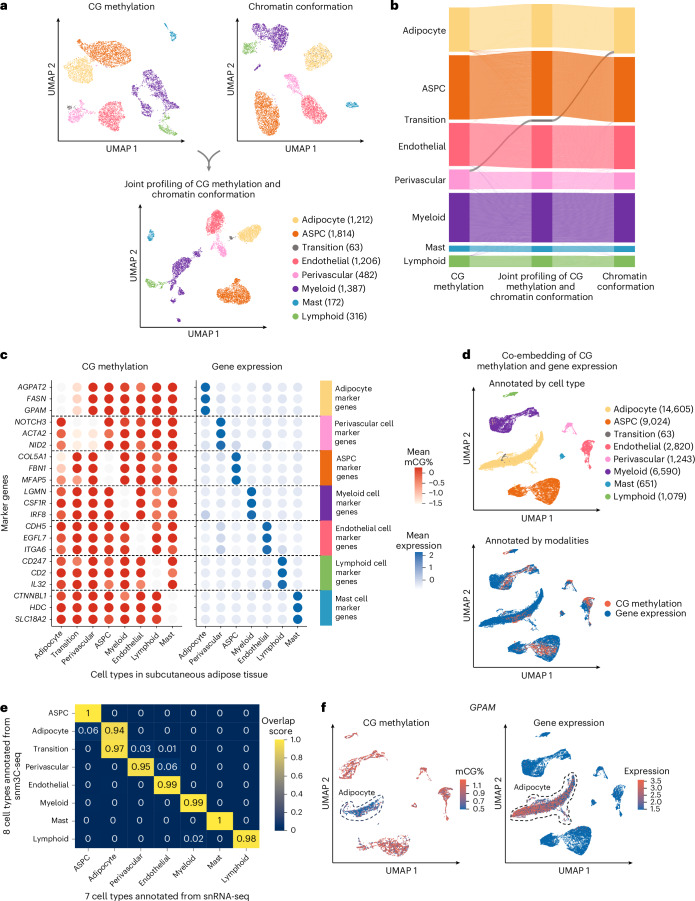
Single-nucleus-level multiomic profiles of SAT by jointly profiling methylation and chromatin conformation with snm3C-seq, followed by an integrative analysis with transcriptomic profiles, generated using SAT snRNA-seq. **a**, Dimension reduction of cells using 5 kb bin mCG (top left), 100 kb bin chromatin conformation (top right) and jointly integrating mCG and chromatin conformation (bottom), profiled by snm3C-seq and visualized with uniform manifold approximation and projection (UMAP). Cells are colored by SAT cell type. **b**, Sankey diagram showcases the high consistency among the SAT cell-type annotations derived from the 5 kb bin mCG (left), 100 kb bin chromatin conformation (right) and joint profiling of mCG and chromatin conformation (middle), with the exception of the transition cell-type cluster that was annotated as perivascular cells by mCG and adipocytes by chromatin conformation. **c**–**f**, Integrative analysis with snRNA-seq, evaluating the concordance of cell-type cluster annotations and cell-type marker genes across modalities. **c**, Comparison of gene body mCG and gene expression profiles of cell-type marker genes across the matching SAT cell types, independently identified within the respective modalities, excluding the expression profiles of the transition cell-type cluster that was not identified in the SAT snRNA-seq data. Dashed lines stratify the marker genes by cell type. Dot colors represent the average gene body mCG ratio normalized per cell (left), and the average log-transformed counts per million normalized gene expression (right). **d**, Co-embedding of cells profiled by snm3C-seq and snRNA-seq. Cells are colored by annotations as in **c** (top) and modalities (bottom). **e**, Concordance matrix comparing the snm3C-seq and snRNA-seq derived annotations, colored by the overlapping scores between the pairs of the SAT cell types evaluated in the co-embedding space. **f**, UMAP visualization of the gene body mCG ratio (left) and gene expression (right) for one adipocyte marker gene, *GPAM*, colored per cell similarly as in **c**. The dashed line highlights the group of cells annotated as adipocytes.

To investigate the cell-type-level epigenomic landscape of human SAT, we profiled DNA methylation, chromatin conformation and gene expression on nuclei isolated from SAT biopsies from Finnish females (Fig. 1 and Supplementary Note 1.1). We used snm3C-seq to simultaneously profile single-cell-level DNA methylation and chromatin conformation of nuclei isolated from five SAT biopsies (see Methods). A total of 6,652 nuclei passed our quality control (Supplementary Fig. 1a,b and Supplementary Note 1.2). We independently identified seven main cell types, including adipocytes, adipose stem and progenitor cells (ASPCs), perivascular, endothelial, myeloid, lymphoid and mast cells, using the global mCG of non-overlapping 5 kb bins and the intrachromosomal contacts among non-overlapping 100 kb bins (Fig. 2a and Supplementary Figs. 2 and 3). Notably, when analyzing the two modalities jointly to derive the de novo snm3C-seq annotation, we discovered a group of nuclei (*n* = 63 nuclei), present in all five samples (constituting the remaining 0.8 ± 0.6%), that demonstrated inconsistent cell-type annotations between the two modalities (that is, categorized as perivascular cells by mCG and as adipocytes by chromatin conformation) (Fig. 2b). We labeled them as a transitional cell-type cluster to highlight their potential developmental stage, observed using the two different omic profiles (Fig. 2a,b).

**Extended Data Fig. 1:**
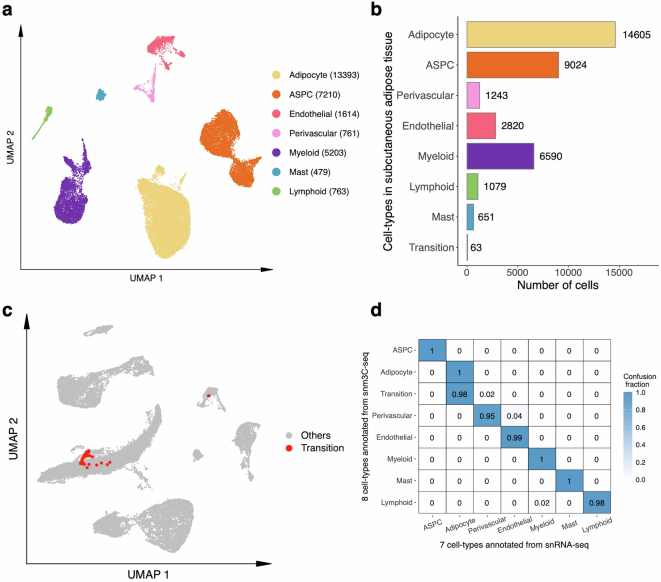
Integrative analysis between subcutaneous adipose tissue (SAT) cells profiled by single-nucleus methyl-3C sequencing (snm3C-seq) and single-nucleus RNA sequencing (snRNA-seq). **a**, Dimension reduction of cells (n = 29,423) profiled by snRNA-seq and visualized with uniform manifold approximation and projection (UMAP). **b**, The total number of cells profiled by snm3C-seq and snRNA-seq stratified by the SAT cell-types. **c**, Co-embedding of snm3C-seq gene body mCG and snRNA-seq gene expression, visualized with UMAP, highlighting the transition cell-type in red and other SAT cell-types in grey. **d**, Confusion matrix comparing the concordance between the *de novo* snm3C-seq annotations (row) and the snRNA-seq-derived annotations (column). The confusion fraction is calculated as the multi-class confusion matrix normalized by the cell counts per row. ASPC, adipose stem and progenitor cell.

**Extended Data Fig. 2:**
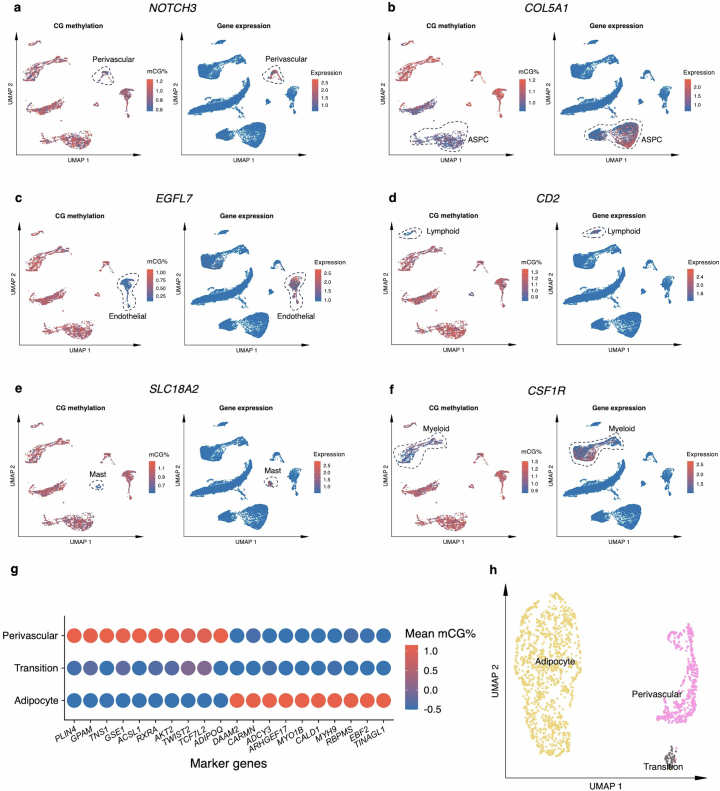
Gene body mCG and RNA expression profiles across SAT cell-type marker genes and clustering analysis of the transition cell-type. **a-f**, Uniform manifold approximation and projection (UMAP) visualization of the gene body mCG ratio, normalized per cell (left) and log-transformed counts per million normalized gene expression (right) for perivascular marker gene *NOTCH3* (**a**), ASPC marker gene *COL5A1* (**b**), endothelial cell marker gene *EGFL7* (**c**), lymphoid cell marker gene *CD2* (**d**), mast cell marker gene *SLC18A2* (**e**), and myeloid cell marker gene *CSF1R* (**f**). The dashed line highlights the group of cells annotated as the corresponding cell-type. **g**, Gene body hypo-methylation of adipocyte marker genes (left 10 columns) and perivascular cell marker genes (right 10 columns) across adipocytes, perivascular cells, and the transition cell-type. Dot colors represent the average gene body mCG ratio normalized per cell. **h**, Dimension reduction of cells profiled by snm3C-seq and restricted to adipocytes, perivascular cells, and the transition cell-type, using exclusively the 5-kb bin mCG profiles and visualized with UMAP. ASPC, adipose stem and progenitor cell.

We next investigated whether SAT snm3C-seq data can be integrated with SAT snRNA-seq data. First, we applied snRNA-seq on nuclei isolated from the same five SAT samples and three additional SAT samples from the Tilkka cohort to obtain 29,423 SAT nuclei (see Methods) and annotated them with matching cell-type resolution (Supplementary Note 1.3, Extended Data Fig. 1a and Supplementary Fig. 1c). Similar to previous brain results^15^, we observed strong and consistent correlations between gene body mCG hypomethylation and RNA expression across the identified cell types (Fig. 2c). This correlation enabled us to integrate and co-embed the snm3C-seq and snRNA-seq cells in the shared canonical component space^19^ (Fig. 2d, Extended Data Fig. 1b,c and Supplementary Fig. 4). Overall, these independently performed modality-specific annotations achieved a ≥0.94 overlap score across all cell-type pairs, in which a higher score indicates better integrated cells in the co-embedding space (see Methods) (Fig. 2e). Comparison between the snm3C-seq de novo annotation and its RNA-derived counterpart resulted in an adjusted Rand index of 0.975 and ≥0.95 confusion fraction (Extended Data Fig. 1d). Unique cell-type marker genes by these two modalities are shown in Supplementary Tables 1 and 2. Taken together, the observed cell-type epigenome profiles, identified using snm3C-seq, exhibit strong concordance with those derived from the snRNA-seq transcriptome; however, at the same time, they carry distinct modality-specific information. For example, the expression of a key adipocyte marker gene, *GPAM*, coincides with demethylation of the gene in the co-embedding space, which may allow for the recruitment of relevant proteins; for example, TFs (Fig. 2f and Extended Data Fig. 2a–f). In summary, mCG and chromatin conformation profiles generated by snm3C-seq robustly recapitulated epigenomic profiles of known major SAT cell types while also uncovering a subtle transition cluster, supporting the differentiation of human adipocytes through a previously less well-characterized route from the perivascular progenitors to adipocytes (Supplementary Note 1.4, Fig. 2, Extended Data Figs. 1 and 2 and Supplementary Figs. 5 and 6).

### Modality-specific and modality-shared molecular mechanisms

**Fig. 3:**
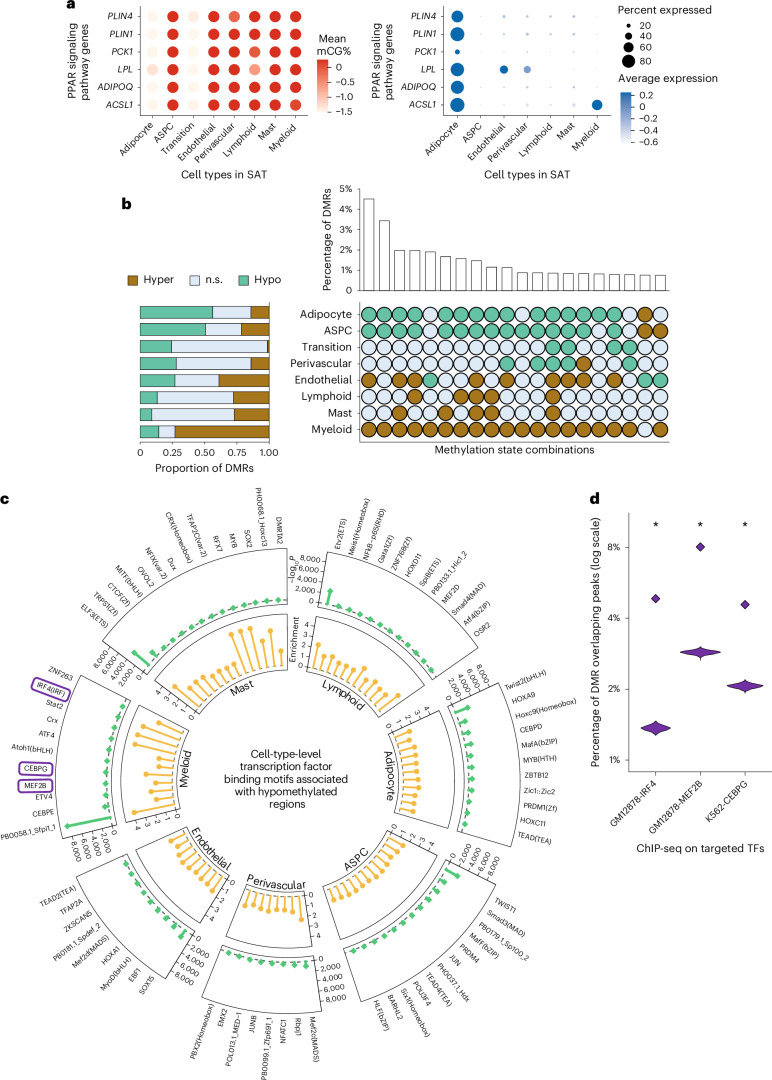
Functional pathways and gene regulatory potential of cell-type-level gene body mCG markers and DMRs. **a**, Dot plots of PPAR signaling pathway genes (*ACSL1*, *ADIPOQ*, *LPL*, *PCK1*, *PLIN1* and *PLIN4*) that are shared adipocyte marker genes between the gene body mCG and gene expression modalities, showing their gene body mCG (left) and gene expression profiles (right) across SAT cell types. The color of the dot represents the mean percentage of mCG (left) and average expression of genes (right), while the size of the dot represents the percentage of cells in which the gene is expressed (right). **b**, Horizontal stacked bar plot (left) showing the marginal proportions of assigned methylation states across DMRs for each SAT cell type (n.s., non-significant) and upset plot (right) displaying the top 20 most prevalent methylation state combinations across all detected DMRs, ordered by decreasing frequency, along with their respective percentages. **c**, Circular plot summarizing the cell-type-level TF binding motifs associated with hypomethylated regions in SAT cell types. Statistical significance was determined using HOMER (binomial test) on each cell type separately. The false-positive rate was calibrated by a stringent cutoff on the unadjusted *P* values (that is, *P* < 1 × 10^−12^). Track 1 shows the negative log *P* values (green lollipops) and track 2 shows the enrichment scores (yellow lollipops). **d**, Violin plots reflecting the empirical null distribution (*n* = 1,000) of the percentages of DMRs overlapping ChIP-seq peaks for three TFs highlighted in **c** (see Methods). Diamonds mark the observed overlapping proportion of the hypomethylated regions in myeloid cells in the corresponding ChIP-seq experiment. Asterisks (*) indicate statistical significance, evaluated based on a one-tailed hypergeometric test for overrepresentation of peaks in the corresponding cell-type-level hypomethylated regions (−log_10_*P* = 646, 1,860 and 1,618 for IRF4, MEF2B and CEBPG, respectively). GM12878, lymphoblastoid cell line; K562, chronic myelogenous leukemia cell line.

**Extended Data Fig. 3:**
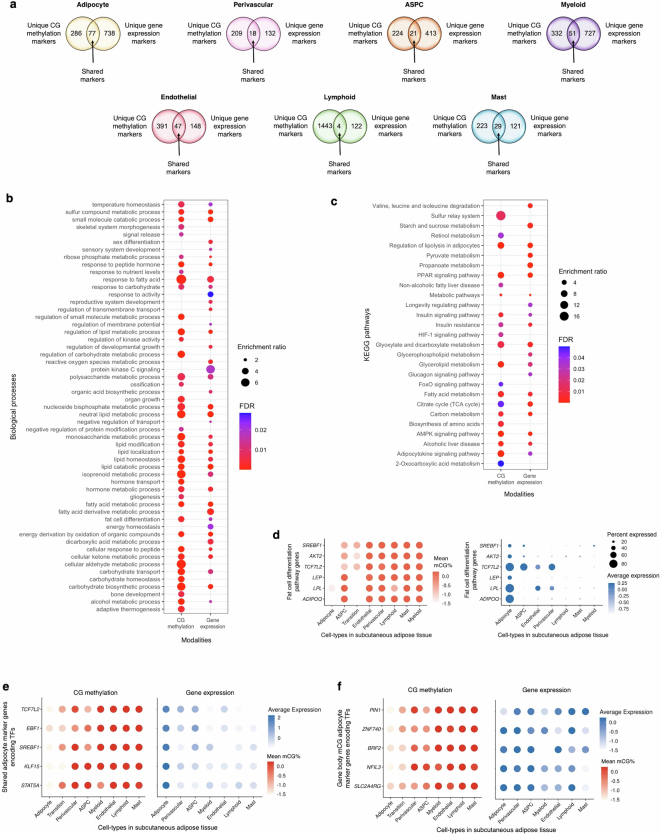
Comparisons of unique cell-type marker genes in SAT cell-types, and biological processes and functional pathways enriched among the adipocyte marker genes between the gene body mCG and gene expression modalities, as well as both known and less-known transcription factors (TFs) in SAT. **a**, Venn diagrams showing the number of shared and modality-specific unique SAT cell-type marker genes between the gene body mCG and gene expression modalities. **b-c**, Dot plots showing significantly enriched biological processes (**b**) and KEGG functional pathways (**c**) using unique adipocyte marker genes in gene body mCG and gene expression modalities, evaluated using WebGestalt (see Methods). *P* values were adjusted for multiple hypothesis testing by the Benjamini-Hochberg procedure. Statistical significance was determined as FDR < 0.05. The size of the dot represents the enrichment ratio for biological processes (**b**) and KEGG functional pathways (**c**), while the color of the dot indicates FDR. **d**, Dot plots of fat cell differentiation biological process genes that are shared adipocyte marker genes between the mCG and gene expression modalities, showing their average gene body mCG (left) and average gene expression profiles (right) across the SAT cell-types. Dot size (right) represents the percentage of cells where the gene is expressed. **e-f**, Dot plots of (e) adipocyte marker genes encoding both known and less known TFs that are shared between the gene body mCG and gene expression modalities and (f) adipocyte marker genes encoding both known and less known SAT TFs that are unique to gene body mCG, showing their gene body mCG (left) and gene expression profiles (right). The color of the dot represents the mean percentage of mCG (red is high) and the average expression of a gene. ASPC, adipose stem and progenitor cell; and FDR, false discovery rate.

Highly expressed SAT cell-type marker genes, revealed by each modality, may elucidate how epigenomic regulation is involved in biological processes and pathways. For both gene body mCG and gene expression, we identified modality-specific and modality-shared marker genes across all SAT cell types (Supplementary Note 1.5 and Extended Data Fig. 3a) and subsequently in shared and non-shared biological processes (Extended Data Fig. 3b) and Kyoto Encyclopedia of Genes and Genomes (KEGG) pathways enriched among adipocyte marker genes (Extended Data Fig. 3c). In adipocytes, for instance, although only 77 marker genes (21% of mCG and 9% of gene expression markers) are present in both modalities, the majority of biological processes (63% of the pathways identified from mCG and 65% from gene expression) and KEGG pathways (65% of the pathways from both mCG and gene expression) are shared (Supplementary Note 1.6). Focusing on genes involved in the well-characterized PPAR signaling pathway, we observed that six genes, *ACSL1*, *ADIPOQ*, *LPL*, *PCK1*, *PLIN1* and *PLIN4*, are hypomethylated in adipocytes and the transition cell type, while being hypermethylated in the rest of the cell types. Correspondingly, their transcriptomic profiles reveal that they are predominantly expressed only in adipocytes, with minimal expression in other SAT cell types (Fig. 3a). Similarly, fat cell differentiation genes *ADIPOQ*, *LPL*, *LEP*, *TCF7L2*, *AKT2* and *SREBF1* are hypomethylated and predominantly expressed in adipocytes compared to the other SAT cell types (Extended Data Fig. 3d). Additionally, modality-shared adipocyte marker genes encode TFs, encompassing both known and less-known regulators critical to SAT (Supplementary Note 1.7 and Extended Data Fig. 3e,f). Collectively, these findings suggest a coordinated regulation of key pathways, biological processes and TFs through both transcriptional and epigenomic mechanisms.

### Striking differences between adipocytes and myeloid DMRs

To delineate the patterns of cell-type-level DNA methylation in SAT, we identified genome-wide DMRs in eight SAT cell types (adipocytes, ASPCs, transition, perivascular, endothelial, myeloid, lymphoid and mast cells) using methylpy^20,21^ (see Methods). Overall, 15.4% of the CG sites are differentially methylated across the SAT cell types, with a total of 705,063 CG DMRs covering 5.39% of the genome. These DMRs have a mean (±s.d.) length of 220 ± 152 bp and consist of an average of 4.5 ± 5.5 differentially methylated sites (DMSs). The large numbers of DMRs we identified in SAT cell types support distinct cell-type-level methylation patterns.

We observed striking genome-wide differences in the number and abundance of hypomethylated and hypermethylated regions among the SAT cell types (Fig. 3b and Supplementary Table 3). In particular, of the total DMRs, 56.3% (*n* = 396,758; −log_10_*P* = 129 using one-tailed *t*-test; see Methods) and 50.6% (*n* = 356,844; −log_10_*P* = 104, one-tailed *t*-test) are hypomethylated in adipocytes and ASPCs, contrasting with only 14.6% (*n* = 102,756) in myeloid cells. Conversely, we observed that up to 73.0% of the DMRs demonstrate a hypermethylation pattern in the myeloid cells (*n* = 514,434; −log_10_*P* = 163, one-tailed *t*-test) versus merely 14.2% in adipocytes and 21.4% in ASPCs. Jointly investigating the differential methylation states across all cell types revealed that 47.3% of the DMRs exhibit opposing profiles between adipocytes, ASPCs and those of the myeloid cells. Taken together, our finding suggests that the widespread repression of regulatory activity in myeloid cells is typically associated with heightened regulatory activity in adipocytes and ASPCs.

### Cell-type-level TF binding motifs

**Extended Data Fig. 4:**
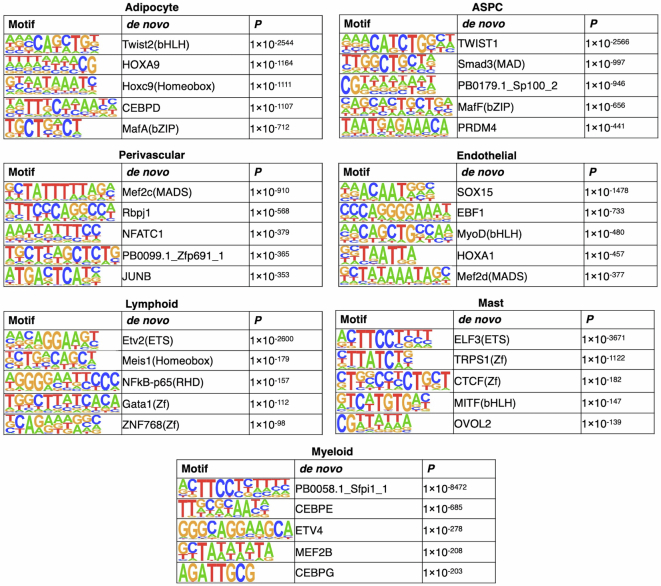
Cell-type level hypo-methylated regions are enriched for specific transcription factor (TF) binding motifs. We show the top five cell-type level TF binding motifs (sorted by *P*) that are enriched among the hypo-methylated regions of the SAT cell-types. Statistical significance was determined using HOMER (binomial test) on each cell-type separately (see Methods). False positive rate was calibrated by a stringent cutoff on the unadjusted *P* values (that is, *P* < 1×10^−12^). ASPC, adipose stem and progenitor cell.

**Extended Data Fig. 5:**
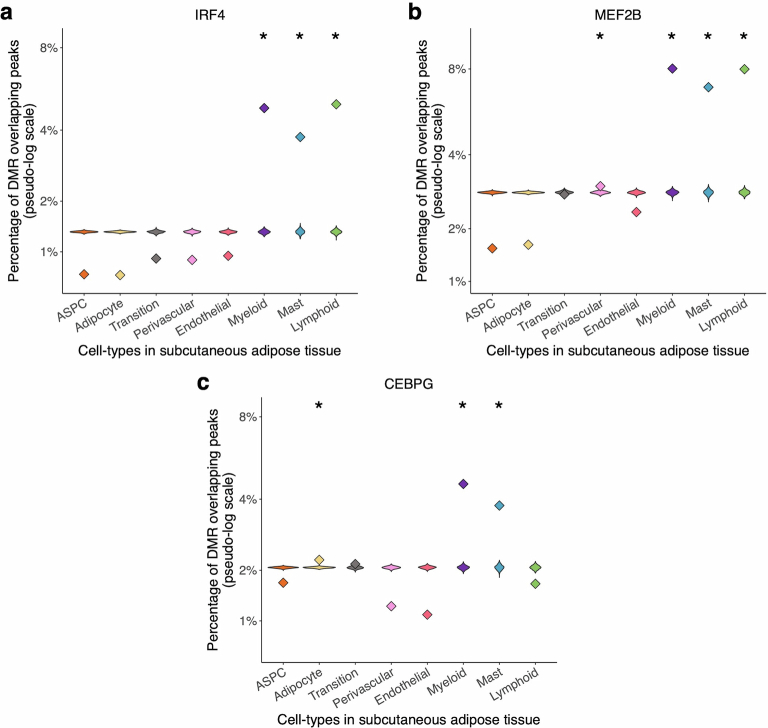
Validation of myeloid-specific transcription factors (TFs), IRF4, MEF2B, and CEBPG, using external ENCODE ChIP-seq data. **a-c**, Violin plots reflecting the empirical null distribution (n = 1,000) of the percentages of DMRs overlapping ChIP-seq peaks in each experiment, stratified by the SAT cell-types. Diamonds mark the observed overlapping proportions of the corresponding cell-type level hypo-methylated regions. *Indicates the statistical significance after multiple hypothesis correction (Bonferroni) on the empirical one-tailed *P* values for the over-representation of TF peaks. IRF4 in GM12878 cell line (empirical unadjusted *P*=0.001 for myeloid, mast, and lymphoid cells) (**a**); MEF2B in GM12878 cell line (empirical unadjusted *P*=0.001 for perivascular, myeloid, mast, and lymphoid cells) (**b**); and CEBPG in K562 cell line (empirical unadjusted *P*=0.001 for adipocytes, myeloid, and mast cells) (**c**). ChIP-seq, Chromatin immunoprecipitation sequencing; ASPC, adipose stem and progenitor cell; GM12878, lymphoblastoid cell line; and K562, chronic myelogenous leukemia cell line.

To investigate the relevance of cell-type-level hypomethylated regions in gene regulation, we performed TF binding motif enrichment analysis using cell-type-level hypomethylated regions. We identified several cell-type-level TFs (Supplementary Note 1.8, Fig. 3c, Extended Data Fig. 4 and Supplementary Table 4), suggesting that these TFs might either bind to the DNA in a cell-type-specific manner or regulate cell-type-level differential methylation patterns. We then sought to validate the cell-type-level TFs that we identified using HOMER^22^ (Fig. 3c) by analyzing external chromatin immunoprecipitation sequencing (ChIP-seq) data from ENCODE^23^ (see Methods). As only immune cell ChIP-seq data are available among the SAT cell types, we focused on validating myeloid-specific TFs (Fig. 3c). As expected, binding peaks for the TFs IRF4 and MEF2B in GM12878 demonstrate 3.5-fold and 2.8-fold enrichments compared to the matching genomic background (empirical *P* < 0.001 for both TFs, −log_10_*P* = 1,618 and 1,860 using one-tailed hypergeometric test, respectively) (Fig. 3d, Extended Data Fig. 5 and Supplementary Table 5). For CEBPG binding peaks in K562, we observed a 2.2-fold enrichment (empirical *P* < 0.001, −log_10_*P* = 647 using one-tailed hypergeometric test) (Fig. 3d, Extended Data Fig. 5 and Supplementary Table 5). Together, these TF enrichments using ENCODE ChIP-seq data in a relevant cell line independently validated our de novo TF results obtained using HOMER (Fig. 3c).

### Enrichment of short-range interactions in SAT adipocytes

**Fig. 4:**
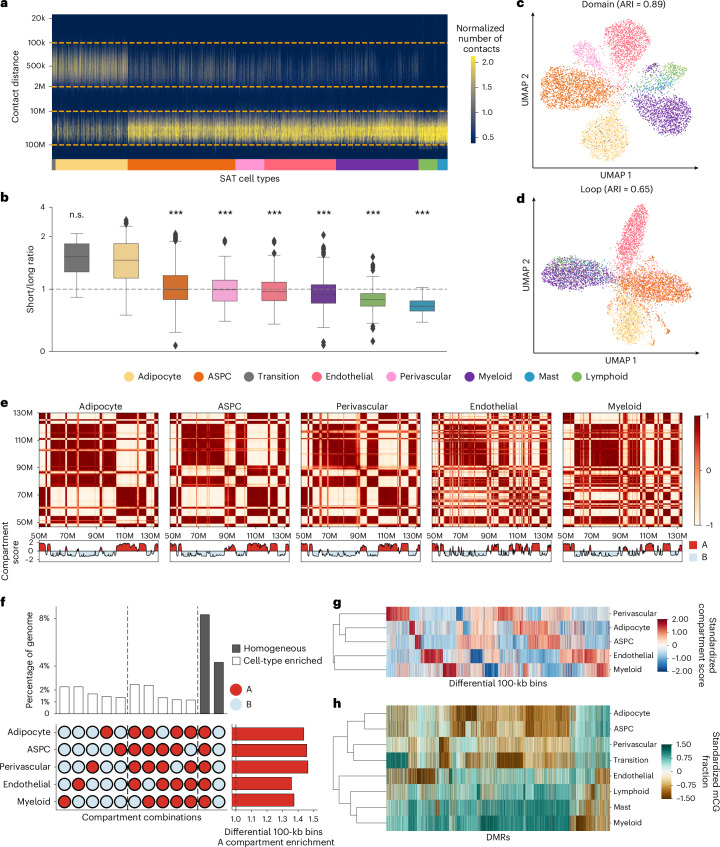
Analysis of chromatin conformation profiles in SAT reveals cell-type-level diversity in compartments, domains and loops. **a**,**b**, Frequency of contacts per cell against genomic distance (*y* axis in log scale), grouped by cell types (**a**) and ordered by the median short-range to long-range interaction ratios (**b**). Dashed lines in **a** mark short-range and long-range contacts. The center of the box in **b** represents the median; the bounds of the box indicate the 25^th^ and 75^th^ percentiles and the whiskers show the minimum and maximum values within 1.5 times the interquartile range. Statistical significance was evaluated based on pairwise one-tailed Wilcoxon rank-sum tests for a higher short-range interaction ratio in adipocytes. Asterisks (***) indicate unadjusted −log_10_*P* > 50. Specifically, unadjusted −log_10_*P* = 197, 116, 221, 258, 128 and 91 for ASPCs, perivascular, endothelial, myeloid, lymphoid and mast cells; *n* = 63, 1,212, 1,814, 482, 1,206, 1,387, 316 and 172 for transition, adipocytes, ASPCs, perivascular, endothelial, myeloid, lymphoid and mast cells, respectively. **c**,**d**, UMAP visualization of low-dimensional embeddings of cells using domains (**c**) and loops (**d**) as features, colored by the snm3C-seq annotation; adjusted Rand index (ARI) evaluates the clustering concordance against snm3C-seq annotation. **e**, Heatmap visualization of the normalized interaction contact map on chromosome 12 and its corresponding compartment scores. **f**, Upset plot (left) visualizing a subset of the differential 100 kb bins and their corresponding percentages; horizontal stacked bar plot (right) showing the marginal A compartment enrichment of differential 100 kb bins. The vertical dotted line separates cell-type-level A compartment flips, B compartment flips and differential bins detected within homogeneous A or B compartments. **g**, Dendrogram of the five most abundant SAT cell types constructed with compartment scores on differential 100 kb bins. **h**, Similar to **g**, except on all annotated SAT cell types, constructed with mCG fractions across DMRs.

**Extended Data Fig. 6:**
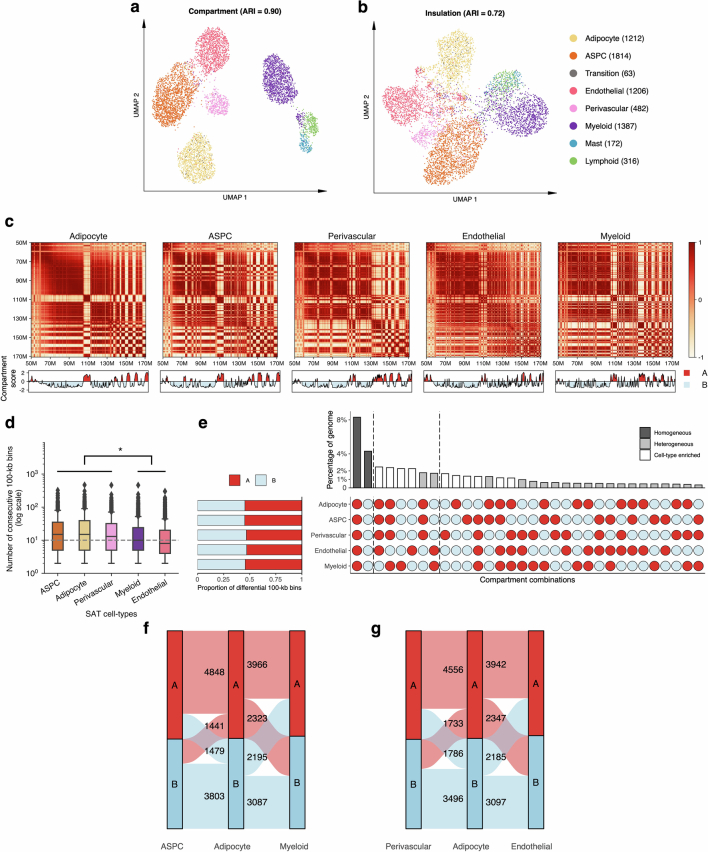
Cell-type level differences in chromatin conformation of subcutaneous adipose tissue (SAT). **a-b**, Uniform manifold approximation and projection (UMAP) visualization of low dimensional embeddings of cells using compartments (**a**) and insulation scores (**d**) as features, colored by the snm3C-seq annotation. Adjusted rand index (ARI) evaluates the clustering concordance against the snm3C-seq annotation. **c**, Heatmap visualization of the normalized interaction contact map on chromosome 6 and its corresponding compartment scores across the SAT cell-types. **d**, Box plots visualizing the distributions of the number of consecutive 100-kb bins within segments that exhibit consistent A or B compartment annotations, stratified by the SAT cell-types. Lone compartments (that is, singular bins, the annotation of which differs from the ones of both neighbors) are excluded. The center of the box represents the median; the bounds of the box indicate the 25% and 75% percentiles, while the whiskers show the minimum and maximum values within 1.5 times the interquartile range. *Indicates unadjusted –log10*P*=25, one-tailed Wilcoxon rank-sum test between the two groups marked by the horizontal line: consistent compartment segments in adipocytes, ASPCs, and perivascular cells (n = 2,890) versus those in myeloid and endothelial cells (n = 2,891). **e**, Horizontal stacked bar plot (left) showing the marginal proportions of differential 100-kb bins stratified by their annotated A and B compartments in the 5 most abundant SAT cell-types and upset plot (right) showing all compartment combinations across differential 100-kb bins in decreasing order with their corresponding percentages. The vertical dotted line separates the following three major categories: ‘Homogeneous’, ‘Cell-type Enriched’, and ‘Heterogeneous’, which correspond to unique A or B compartments in 0, 1, or more than 1 cell-type, respectively. **f**, Sankey diagram breaking down the numbers of differential 100-kb bins annotated as A (red) and B (blue) compartments belonging to ASPCs (left), adipocytes (middle), and myeloid cells (right). **g**, Similar to (f), except on perivascular cells (left), adipocytes (middle), and endothelial cells (right). ASPC, adipose stem and progenitor cell.

Tight packaging of DNA inside the nucleus leads to physical contacts between genomic regions, which affects the gene expression machinery^24^. We observed substantial differences in the distribution of closely and distantly located interaction contacts at various genomic distances across the cells profiled by snm3C-seq (see Methods). In pairwise comparisons with other cell types, adipocytes harbor significantly higher proportions of short-range interactions (100 kb to 2 Mb) compared to long-range interactions (10–100 Mb), with the exception of the transition cell type (−log_10_*P* > 91; one-tailed Wilcoxon rank-sum test) (Fig. 4a,b and Supplementary Note 1.9). This observation is in line with all cell-level clustering analyses using chromatin conformation information at various resolutions, which indicate that the transition cell type shares more similarity with adipocytes (Figs. 2b and 4c,d and Extended Data Fig. 6a,b).

In addition, the observed differences in the ratio of short-range to long-range interactions between ASPCs and adipocytes (one-tailed Wilcoxon rank-sum −log_10_*P* *>* 197) could suggest a link between the contact distances and functionally important genomic regions in adipogenesis. Given that ASPCs develop into adipocytes, we speculate that this change may reflect the unilocular lipid droplet formation in adipocytes that makes them larger than ASPCs.

### Chromosomal conformation dynamics reflect cell-type lineage

Chromatin compartments, which connect stretches of the genome that are tens of megabases apart, reflect how cells arrange their chromosomal structures in three-dimensional space at the highest level^25^. We started our investigation of aggregated SAT cell-type-level genome spatial topology by calculating the compartment scores on the pseudobulk contact matrices for the five most abundant cell types (adipocytes, ASPCs, endothelial, perivascular and myeloid cells) at 100 kb resolution. Based on the sign of the compartment scores, we partitioned the genome into either the active A compartment regions or the more repressive B compartment regions. The correlation matrices derived from the normalized interaction contact maps revealed visually distinct cell-type-level plaid patterns. For example, on chromosome 6 (Extended Data Fig. 6c) and chromosome 12 (Fig. 4e), endothelial and myeloid cells harbor more intricate structures, indicated by the frequent compartment switches, whereas adipocytes, ASPCs and perivascular cells tend to have longer stretches of region being annotated as the same compartment (Supplementary Note 1.10 and Extended Data Fig. 6d).

Upon a closer inspection, a total of 11,571 100 kb bins, spanning 44.3% of the genome, are statistically differentially conformed among the five cell types at a false discovery rate (FDR) < 0.1 cutoff. The empirical FDR is estimated to be ≤0.02 (see Methods). Although each cell type has its own distinct pattern of chromosomal compartments, differential 100 kb bins consistently demonstrate at least a 1.36-fold enrichment landing in the active A compartments compared to the genome-wide background (Fig. 4f, Extended Data Fig. 6e and Supplementary Table 6). Interestingly, across all investigated SAT cell types, the two leading predominant compartment combinations are the homogeneous A and B. These combinations account for 18.8% and 9.7% of the total differentially conformed regions (8.3% and 4.3% of the genome), suggesting significant heterogeneity within each compartment stratification (that is, A and B). These are followed by combinations driven by myeloid and endothelial cells, either through cell-type-level compartment flips (A→B or B→A) or coordinated flips involving both cell types (Supplementary Note 1.10 and Extended Data Fig. 6e–g). Given that we observed similar distinct mCG patterns for endothelial and myeloid cells, we aimed to confirm whether the lineage dendrogram constructed from differential 100 kb bins would mirror the developmental trajectory inferred from DMRs. Indeed, hierarchical clustering consistently grouped endothelial and myeloid cells, characterized by pronounced hypomethylation and frequent compartmental switches, into a distinct branch in both modalities (Fig. 4g,h), in line with a previous report showing that myeloid progenitors also give rise to vascular endothelial cells^26^.

### Cell-type specificity in regional 3D genome structures

**Extended Data Fig. 7:**
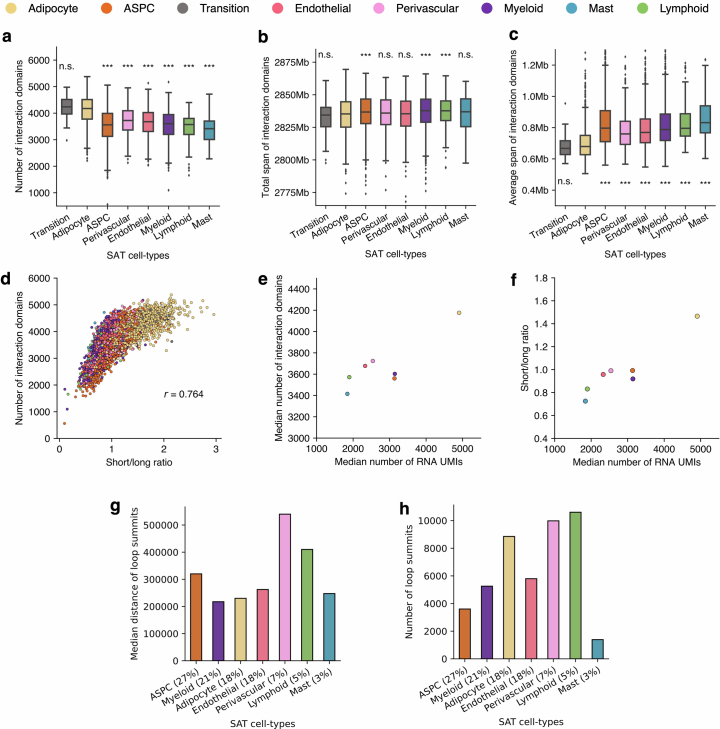
Cell-type specificity in interaction domains and loops. **a-c**, Box plots visualizing the distribution of the number of interaction domains (**a**), the total number (**b**), and the average span (**c**) of interaction domains detected in each cell, stratified by cell-types. The center of the box represents the median; the bounds of the box indicate the 25% and 75% percentiles, while the whiskers show the minimum and maximum values within 1.5 times the interquartile range. Statistical significance was evaluated using pairwise one-tailed Wilcoxon rank-sum tests against adipocytes. *** Indicates Bonferroni multiple hypothesis adjusted *P* < 0.05 and n.s. denotes non-significant. Specifically, the unadjusted -log_10_*P*=133, 45, 92, 132, 70, and 50 for ASPCs, perivascular, endothelial, myeloid, lymphoid, and mast cells, respectively in (**a**); the unadjusted -log_10_*P*=5, 7, and 4 for ASPCs, myeloid, and lymphoid cells, respectively in (**b**), and the unadjusted -log_10_*P*=132, 45, 90, 132, 71, 50 for ASPCs, perivascular, endothelial, myeloid, lymphoid, and mast cells, respectively in (**c**). Each cell is treated as an independent replicate; thus n = 1,212, 1,814, 63, 1206, 482, 1,387, 172, and 316 for adipocytes, ASPCs, transition, endothelial, perivascular, myeloid, mast, and lymphoid cells, respectively. **d**, Scatter plot showing the short to long-range interaction ratio per cell against the number of interaction domains detected. Cells are colored by their snm3C-seq annotation. **e-f**, Scatter plots showing the aggregated cell-type level median number of unique molecular identifiers (UMIs) from snRNA-seq against the median number of interaction domains (**e**) and the ratio of short to long-range interaction contacts (**f**) from snm3C-seq, colored similarly as in **d**. **g-h**, Bar plots showing the median distance (**g**) and the total number (**h**) of loop summits detected across the SAT cell-types (x-axis is ordered by the abundance of each cell-type in snm3C-seq). ASPC, adipose stem and progenitor cell.

In addition to compartmentalization, the genome maintains its finer spatial structure by forming interaction domains and cohesion-mediated chromatin loops. Analyzing snapshots of the 3D genome at 25 kb and 10 kb resolution allowed us to delineate these regional features at both cell and aggregated cell-type resolution. In addition to the transition cell type, adipocytes showcase an accumulation of significantly denser interaction domains (an average of 4,120 per cell, compared to 3,574 in others; −log_10_*P* > 45, pairwise one-tailed Wilcoxon rank-sum test), while spanning a much shorter distance (median of 679,121 bp per cell, compared to 783,213 bp in others; Extended Data Fig. 7a–c). Interestingly, the number of detected domains is highly correlated with the ratio of short-range to long-range interactions (Pearson’s correlation coefficient, 0.76; Extended Data Fig. 7d). This observation reinforces the idea that regional contacts are necessary to support the more intricate local 3D structures. Both features correlate with the general transcriptomic activity in the matching snRNA-seq data, in which adipocytes show a 1.5-fold increase in the total number of unique molecular identifiers (Extended Data Fig. 7e,f). Overlapping cell-type pseudobulk insulation scores with boundary probability, calculated as the fraction of cells with a boundary detected in a given cell type, identified a total of 1,791 differential boundaries (see Methods). Regarding chromatin loops, we detected a median of 47,837 and 5,797 cell-type-level loop pixels and merged loop summits, respectively. Adipocytes demonstrate a similar trend of having more loop summits (*n* = 8,852) and a relatively shorter median loop length (230,000 bp, compared to others 290,000 bp; Extended Data Fig. 7g,h). Along with the clustering results derived from regional interaction features (for example, insulation scores, domains and loops), which show highly concordant annotations (Fig. 4c,d and Extended Data Fig. 6a,b), we conclude that granular 3D genomic features also exhibit significant heterogeneity across SAT cell types.

### Influence of 3D topology on gene regulatory landscapes

**Extended Data Fig. 8:**
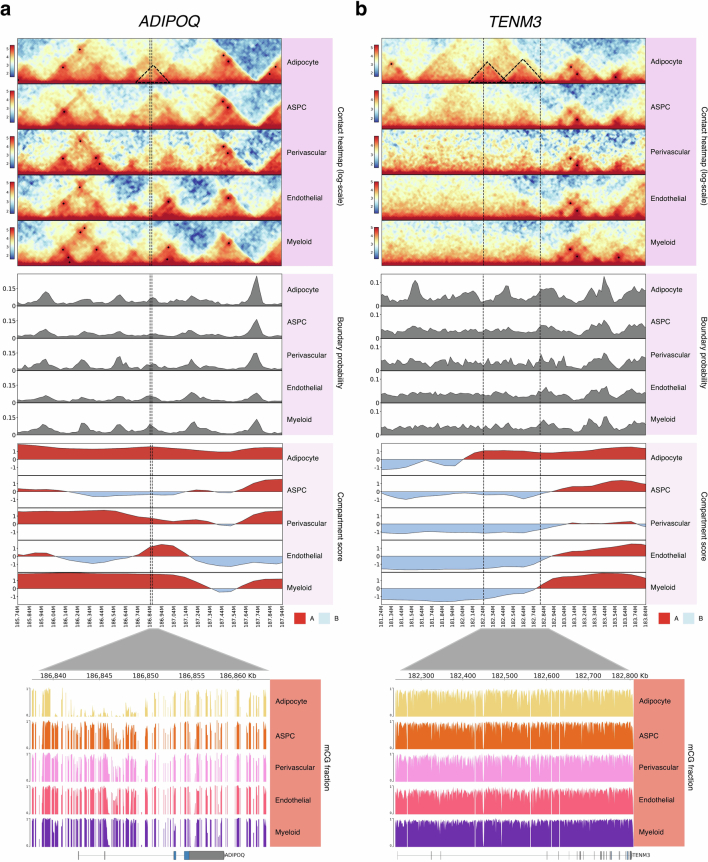
Browser-based stacked views of multimodal data for adipocyte marker genes in five major SAT cell-types. **a-b**, From top to bottom: chromatin conformation contact heatmap, imputed at a 10-kb resolution and colored at log-normalized scale; boundary probabilities calculated at a 25-kb resolution; and chromosome compartment scores evaluated at a 100-kb resolution, where the A compartments are colored red and the B compartments blue. The shaded trapezoid connects the top panels to a zoomed-in view of the CG methylation patterns surrounding the gene body, extending 5-kb up- and downstream. Vertical dashed lines mark the gene start and end positions. Black dots represent the detected chromatin loop summits. The black dashed triangles in the contact heatmap highlight differential domains that are present only in adipocytes: one encapsulates the *ADIPOQ* gene (**a**), and two reside within the *TENM3* gene body (**b**). SAT, subcutaneous adipose tissue; and ASPC, adipose stem and progenitor cell.

The 3D topology of a cell also influences its transcriptomic dynamics with cell-type specificity. As expected, genes expressed in a cell tend to localize in its active A compartment, exhibiting ≥2.24-fold enrichment relative to the B compartments across the five most abundant cell types. These ratios increase when restricted to the set of cell-type-level unique marker genes. Perivascular cells, in particular, exhibit a staggering 13-fold A/B ratio, leading to 92.8% of the unique marker genes landing in the A compartments (Supplementary Table 7). We next focused on ASPCs, a cell type that undergoes active differentiation into adipocytes, and systematically evaluated how compartment flipping affects the downstream expression. On a global scale, 23.4% of the differentially conformed regions change from the ASPC A compartments to adipocyte B, while 27.5% convert from the B compartments to A. Residing within these topologically interesting regions are some key cell-type marker genes crucial for adipogenesis (Supplementary Note 1.11). For example, *ADIPOQ* is located in an interaction domain unique to adipocytes with a pronounced demethylation pattern around its gene body, probably facilitating additional TFs to bind and activate it functionally. The adjacent differential boundary marks a stretch of the genome, encapsulating 1 Mb upstream and downstream of *ADIPOQ*, that transitions from the inactive B compartments in ASPCs to the active A compartments in adipocytes (Extended Data Fig. 8a). Other strong gene expression de novo adipocyte marker genes, including *TENM3*, *CSMD1* and *PCDH9*, also land within differential domains specific to adipocytes; additionally, *CSMD1* and *PCDH9* have cell-type-level loop domains near the transcription start sites (Extended Data Fig. 8b and Supplementary Fig. 7a,b). Together, our findings suggest that chromosome conformation, ranging from mega-base compartmentalization to kilo-base loop formation, reflects a higher level of carefully balanced coordination among the SAT cell types, influencing both their epigenetic regulation profiles and trickling down to their transcriptional activities.

### Contribution of *DNMT3A* and *TET1* to DMR patterns

**Fig. 5:**
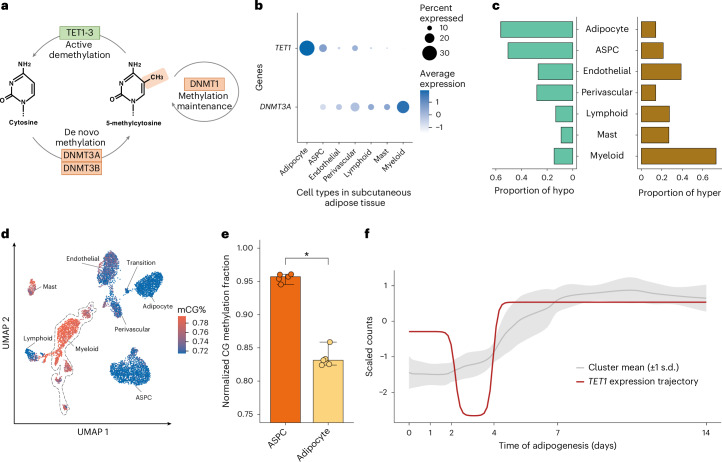
Analysis of mean gene expression and DMRs across SAT cell types reveals the potential involvement of DNA methylation pathway genes in regulating cell-type-level hypermethylation and hypomethylation in SAT. **a**, A schematic representation of basic mechanisms and key players in DNA methylation and demethylation. **b**, Dot plot of *TET1* and *DNMT3A* showing their expression profiles across the SAT cell types. The size of the dot represents the percentage of cells in which a gene is expressed within a cell type, and the color represents the average expression of each gene across all cells within a cell type (blue indicates higher expression). **c**, Proportions of assigned hypomethylated (left) and hypermethylated states (right) across DMRs. **d**, UMAP visualization of the average global mCG ratio in a cell. The dashed line highlights those annotated as myeloid cells. **e**, Bar plot reflecting the distribution of normalized mCG fractions across genes that co-clustered with *TET1* in **f** for ASPCs and adipocytes. Asterisk (*) indicates statistical significance from a paired one-tailed Wilcoxon rank-sum test, comparing the median cluster expression across *n* = 5 samples, showing higher expression in ASPCs than in adipocytes (unadjusted *P* = 0.031). The center of the bar represents the median, and the error bars represent the highest and lowest expression across *n* = 5 samples. **f**, Longitudinal expression of *TET1* is plotted across the 14-day SAT preadipocyte differentiation. The shaded ribbon behind the trajectory of *TET1* reflects the mean and standard deviation of the genes that clustered into similar trajectory patterns as *TET1* using DPGP.

**Extended Data Fig. 9:**
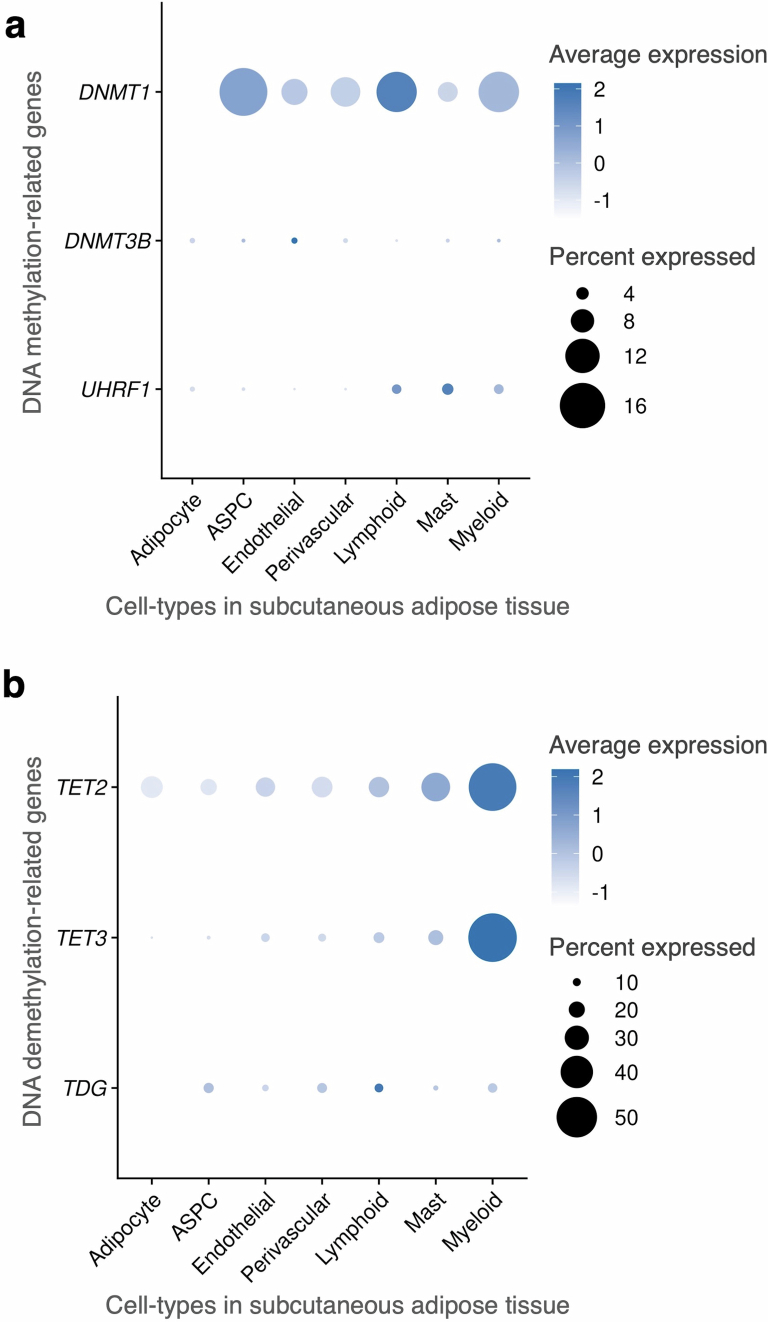
Mean gene expression of DNA methylation- and demethylation-related genes across the cell-types in subcutaneous adipose tissue (SAT). **a-b**, Dot plot showing expression of (**a**) DNA methylation genes (*DNMT1, DNMT3B*, and *UHRF1*) and (**b**) DNA demethylation genes (*TET2*, *TET3*, and *TDG*) across SAT cell-types. The size of the dot represents the percentage of cells in which a gene is expressed within a cell-type, while the color represents the average expression of each gene across all cells within a cell-type (blue indicates a higher expression). ASPC, adipose stem and progenitor cell.

DNA methylation involves the covalent addition of a methyl group to DNA, a process facilitated by DNA methyltransferase enzymes, such as DNA methyltransferase 3 alpha (DNMT3A) and DNA methyltransferase 3 beta (DNMT3B)^27^. Conversely, DNA demethylation comprises the removal of this methyl group from the DNA by the ten-eleven translocation (TET) family proteins, specifically, TET1, TET2 and TET3 (Fig. 5a). Among the demethylase genes, *TET1* is preferentially expressed in adipocytes (−log_10_*P* > 300; two-sided Wilcoxon rank-sum test) (Fig. 5b), in line with our observation that adipocytes have significantly more hypomethylated (56.3%) than hypermethylated regions (14.2%) (Fig. 5c,d). Among the DNA methyltransferases, our cell-type-level SAT snRNA-seq data show that *DNMT3A* is predominantly expressed in myeloid cells, with minimal or no expression in adipocytes (−log_10_*P* = 136; two-sided Wilcoxon rank-sum test) (Fig. 5b). Consistent with our *DNMT3A* expression results, 73.0% of DMRs are hypermethylated in the myeloid cells while only 14.2% are hypermethylated in adipocytes (Fig. 5c). In addition, the expression of a methylation maintenance gene, *DNMT1*, is significantly lower in adipocytes than other SAT cell types (−log_10_*P* = 170; two-sided Wilcoxon rank-sum test) (Extended Data Fig. 9a). Expression of other methylation (*DNMT3B* and *UHRF1*) and demethylation genes (*TET2, TET3* and *TDG*), shown in Extended Data Fig. 9a,b, suggest that *TET1* and *DNMT3A* are the most important genes that contribute to the observed adipocyte hypomethylation and myeloid hypermethylation patterns, indicating their potential mechanistic role in cell-type-level DNA methylation signatures in SAT. The SAT bulk RNA-seq dataset further revealed that bulk expression of *DNMT3A* is negatively associated with insulin sensitivity using the Matsuda Index, whereas bulk expression of *TET1* is positively correlated with the Matsuda Index (Supplementary Note 1.12 and Supplementary Table 8). In addition, we observed that *TET1* is temporally co-expressed with known adipogenesis genes across human primary preadipocyte differentiation (Supplementary Note 1.13, Fig. 5e,f and Supplementary Table 9). Taken together, the cell-type-level expression patterns of *DNMT3A* in myeloid cells and *TET1* in adipocytes suggest that their association signals with CMD traits we observed in the SAT bulk expression may largely originate from their high expression in these two specific cell types.

### Variants in DMRs and compartments are enriched for CMD risk

We next explored the genetic contributions to key CMDs relevant to SAT from variants residing in the cell-type-level compartments and DMRs. Specifically, we assessed abdominal obesity using waist-hip-ratio adjusted for body mass index (WHRadjBMI) as its well-established proxy^28,29^, body mass index (BMI), CRP and metabolic dysfunction-associated steatotic liver disease (MASLD) in the UK Biobank (UKB)^30,31^.

**Fig. 6:**
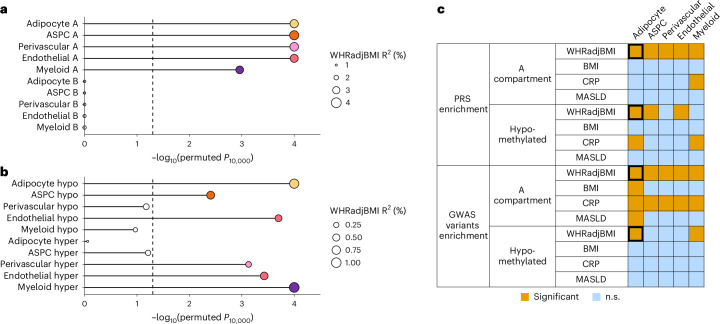
Partitioned PRS and GWAS enrichment results for four key cardiometabolic traits, stratified by cell-type-level DMRs and compartments. **a**,**b**, Lollipop plots depict the incremental variance explained of each cell-type-level PRS for abdominal obesity (using WHRadjBMI as a proxy) from the (**a**) A and B compartments and (**b**) hypomethylated and hypermethylated regions. Each lollipop represents a WHRadjBMI PRS, in which dot size corresponds to the incremental variance explained of the PRS. Significant enrichment of incremental variance explained was determined empirically by comparing to *n* = 10,000 permutated PRSs (see Methods). The gray vertical dashed line indicates the significance cutoff based on unadjusted one-tailed permutation *P* values (that is, *P*_perm_10,000 < 0.05). Bars and lollipops corresponding to cell types with significantly enriched PRSs are colored by cell type, whereas those without are outlined in gray without a filling. **c**, Summary table visualizing the overall PRS and GWAS enrichment results across four key cardiometabolic traits (that is, WHRadjBMI, MASLD, BMI and CRP), stratified by cell-type-level hypomethylated regions and A compartments. Orange indicates nominal significance (*P* < 0.05) on the one-tailed permutation *P* values for PRSs and the one-tailed hypergeometric *P* values for GWAS enrichment. The bolded black border highlights the following consistently significant cell-type-cardiometabolic trait pair: adipocyte–WHRadjBMI.

**Extended Data Fig. 10:**
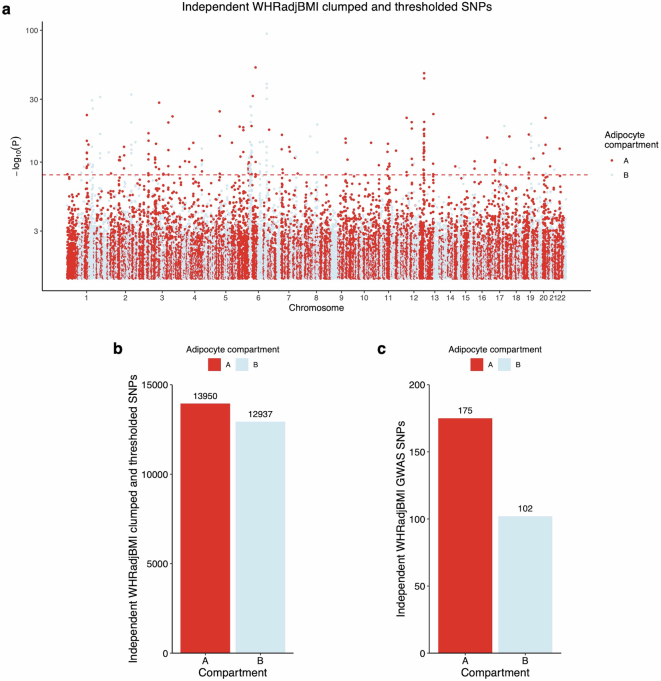
Abdominal obesity -associated variants are enriched for the adipocyte A compartment. **a**, The clumped and thresholded variants (R^2^ < 0.2, unadjusted *P* < 0.05) used for constructing the adipocyte compartment PRSs for abdominal obesity (employing WHRadjBMI as a proxy) are plotted by genomic position against the unadjusted -log_10_*P* from the UK Biobank WHRadjBMI GWAS (n = 195,863 unrelated Europeans). SNPs landing in the adipocyte A compartments are colored red, while SNPs landing in the adipocyte B compartments are colored blue. The horizontal dashed line indicates genome-wide significance (unadjusted *P* < 5 × 10^−8^). **b**, Bar plot showing the number of WHRadjBMI-associated variants in (**a**), stratified by the adipocyte compartment assignment. **c**, Similar to (**b**) except showing the number of independent (R^2^ < 0.2) WHRadjBMI GWAS variants, passing genome-wide significance (unadjusted *P* < 5 × 10^−8^). PRS, polygenic risk score; WHRadjBMI, waist-hip-ratio adjusted for body mass index; and SNP, single nucleotide polymorphism.

We first evaluated the partitioned polygenic risk of variants landing in cell-type-level compartments and DMRs (see Methods). Among the four traits, we highlight WHRadjBMI. All WHRadjBMI polygenic risk scores (PRSs) built from variants in the cell-type-level A compartments are significant (*P*_R_^2^ < 0.05) and enriched predictors (*P*_perm10,000_ < 0.05), explaining ≥80% of variance in WHRadjBMI captured by the full genome (Fig. 6a, Extended Data Fig. 10a,b and Supplementary Table 10). Conversely, we observed no PRS enrichment from the B compartments. We also observed enrichment of the WHRadjBMI polygenic risk from variants in cell-type-level DMRs and strong genetic contributions to CMDs from the target eGenes of the SAT *cis*-expression quantitative trait locus variants, residing in these cell-type-level DMRs using HuGE scoring^32^ (Supplementary Note 1.14, Fig. 6b and Supplementary Tables 11 and 12). Additionally, we found that PRSs constructed from the A compartments and the hypomethylated regions of myeloid cells are enriched predictors of CRP, suggesting a low-grade inflammatory role for this immune cell type (Supplementary Note 1.14, Fig. 6c and Supplementary Tables 10 and 11).

Next, we examined the cell-type-level compartments and DMRs for overrepresentation of independent GWAS variants for the four outcomes. We observed that all five cell-type-level A compartments are enriched for both WHRadjBMI and CRP GWAS variants (Fig. 6c, Extended Data Fig. 10c and Supplementary Table 13). Adipocyte A compartments are additionally enriched for BMI and MASLD GWAS variants. For DMRs, we observed enrichment of WHRadjBMI GWAS variants in adipocyte and myeloid hypomethylated and in ASPC and perivascular hypermethylated regions, as well as strong genetic contributions to obesity-related outcomes from their adjacent genes using HuGE scoring (Supplementary Note 1.14, Fig. 6c and Supplementary Table 14).

Overall, our results highlight the SAT cell-type-level methylation and spatial conformation profiles as important contexts underlying the genetic risk of CMDs, with the hypomethylated regions and the active A compartments of adipocytes giving the strongest signal for abdominal obesity risk and the A compartments of myeloid cells more indicative of inflammation risk.

## Discussion

Delineating the cell-type-level epigenomic landscape in human SAT is crucial for understanding its regulatory mechanisms and impact on obesity risk. Our data revealed a highly dynamic reciprocal interplay between the SAT cell-type-level epigenomes, particularly between adipocytes and myeloid cells. We further integrated the differential epigenomic sites with variant-level data in the UKB, thus uncovering their significant contributions to the genetic risks of CMDs, most notably abdominal obesity. Finally, we elucidated the potential role of specific methylation and demethylation pathway genes in the cell-type-level differential methylation of human SAT.

Focusing on bulk methylome profiles, previous studies have reported differential DNA methylation patterns at the tissue level in SAT and their association with obesity^33,34^. However, underlying non-captured cell-type-level methylation patterns and composition often confound tissue-level analyses^35^. Our SAT cell-type-level methylation profiles and DMRs could serve as reference panels and provide informative features for computationally decomposing the heterogeneous SAT mixtures^36–38^, a critical step for reducing false discoveries in tissue-level studies and facilitating cell-type-specific biomarker identification^36,39–42^. The cell-type composition itself could also hold substantial clinical implications. For example, previous research using transcriptome profiling of perigonadal adipose tissue in mice and immunohistochemistry of human SAT has suggested that the accumulation of myeloid cells, particularly macrophages, correlates with increased adiposity^43^.

Among the identified cell-type-level 3D genome structures in SAT, adipocytes showcase a distinct regional topology, with a 1.51-fold enrichment in relative short-range interactions, a 1.15-fold increase in the number of domains and a 1.72-fold increase in overall transcriptomic activity. Similar patterns have been observed in other human solid tissues, and notably, these types of differences in the non-neuronal cells in the brain have been linked to larger nuclear size^10,44,45^. The presence of the key adipocyte marker gene and adipokine *ADIPOQ* in a genomic region differentially conformed between ASPCs and adipocytes while heavily demethylated in adipocytes further supports the idea that epigenomic structures reorganize during cell differentiation^46^, ultimately regulating downstream, regional and cell-type-level gene expression.

In our cell-type-level investigations of methylation pathway genes, we found notably high expression of *TET1* in adipocytes and *DNMT3A* in myeloid cells, supporting a tissue and cell-type-level reciprocal coordination and cross-talk between *TET1* expression and hypomethylation in adipocytes and *DNMT3A* expression and hypermethylation in myeloid cells. A previous study showed that *TET1* is an important DNA demethylase in adipose bulk tissue^47^. An earlier study reported the involvement of *TET1* in adipocytokine promoter hypomethylation in adipocytes^48^. Furthermore, prior studies have also demonstrated that both *TET1* and *DNMT3A* compete to regulate epigenetic mechanisms^49^. Although previous studies have investigated *TET1* (refs. ^47,50,51^) and *DNMT3A*^52,53^ in adipose bulk tissue level or some of the SAT cell types, we investigated their expression at the single-cell level in all main SAT cell types from the human SAT biopsies, thus providing more granular information about these key methylation genes.

Abdominal obesity is highly polygenic^54^. Previous studies have successfully built predictive genome-wide PRSs for abdominal obesity^55,56^ and shown that a high genetic predisposition to abdominal obesity predicts the regain of abdominal obesity following weight loss^54^. However, less is known about the characteristics of specific genomic regions that contribute most to the polygenic risk of abdominal obesity, which could ultimately improve individual disease risk assessment. By constructing the partitioned PRSs and examining the presence of the independent strongest GWAS variants for abdominal obesity based on the two epigenomic modalities, we discovered that the GWAS variants landing in both the hypomethylated regions and those in the active A compartments of adipocytes show robust associations for abdominal obesity.

Our study has some limitations. First, even though snm3C-seq reveals both intrachromosomal and inter-chromosomal interactions, we have only presented extensive analyses of the former. Although we recognize the potential interest in inter-chromosomal interactions, single-cell 3D genome data currently remains relatively sparse and does not yet support robust and reliable identification of inter-chromosomal interactions^10,12,16,17^. Second, generating snm3C-seq data in frozen human solid tissues remains time-consuming and expensive, requiring relatively large biopsies compared to other single-cell omics platforms. As a result, this study only includes Finnish females without obesity. Inclusion of males and individuals with obesity would help elucidate potential sex-specific epigenomic landscapes and differences across the various BMI categories. Similarly, larger sets of samples from additional populations are also warranted. Third, inclusion of visceral adipose tissue data into future studies would uncover cell-type-level methylation and chromatin conformation patterns in this other metabolically important adipose depot as well as their differences compared to SAT. Nevertheless, taken together, our study provides a valuable insight into the cell-type-level epigenomes in human SAT to be followed up in future studies.

## Methods

### Ethics

The Tilkka study was approved by the Helsinki University Hospital Ethics Committee (approval no. HUS/1039/2019). The UKB study was approved by the North West Multi-centre Research Ethics Committee (approval no. 21/NW/0157). The Finnish METabolic Syndrome In Men (METSIM) study was approved by the Ethics Committee of the Northern Savo Hospital District (approval no. 171/2004). All participants provided written informed consent. All research conformed to the principles of the Declaration of Helsinki. No compensation was provided to the participants.

### Tilkka cohort

Eight Finnish females without obesity underwent abdominal SAT liposuction at Tilkka Hospital, Helsinki, Finland. We performed snRNA-seq on all eight SAT biopsies and snm3C-seq on five SAT biopsies. The mean age (±s.d.) for the snRNA-seq cohort was 44.5 ± 6.03 years, and for the snm3C-seq cohort, the mean age was 44.4 ± 4.78 years.

### UKB cohort

For our GWAS enrichment and PRS analyses, we used genotype and phenotype data from 391,701 unrelated individuals (54.0% female; mean age, 56.9 ± 8.0 years) of European origin from the UKB^30,31^. Data for UKB were collected across 22 assessment centers^30,31^. Genotype data were obtained using either the Applied Biosystems UK BiLEVE Axiom Array or Applied Biosystems UKB Axiom Array and imputed with the Haplotype Reference Consortium and the merged UK10K and 1000 Genomes phase 3 reference panels^30,31^. Data from UKB were accessed under application 33934.

### METSIM cohort

Unrelated men (*n* = 335; mean age, 54.1 ± 4.9 years) from the METSIM cohort^57^ underwent SAT biopsies for bulk RNA-seq. These METSIM participants were recruited from the University of Eastern Finland and Kuopio University Hospital, Kuopio, Finland.

### In situ chromatin conformation capture and fluorescence-activated nuclei sorting

We performed in situ chromatin conformation capture using an Arima Genomics Arima-HiC Kit^45^ with the following modification: the amount of Triton-X 100 in the NIBT buffer was increased to 1% to account for the large amount of fat in adipose tissue. Fluorescence-activated nuclei sorting and library preparation were performed using the snmC-seq3 workflow (https://www.protocols.io/view/snm3c-seq3-kqdg3x6ezg25). The snmC-seq3 libraries of human SAT were sequenced using an Illumina NovaSeq 6000 instrument with S4 flow cells, generating 150 bp paired-end reads. The sequencing reads of snm3C-seq were mapped using Taurus-MH^12^ (https://github.com/luogenomics/Taurus-MH).

### Snm3C-seq quality control and preprocessing

We filtered the cells profiled by snm3C-seq based on the following metrics^10,12^: (1) estimated non-conversion rate of mCCC% < 0.015; (2) global mCG% > 0.5; (3) global mCH% < 0.15; (4) total number of interaction contacts >100,000 and < 500,000; and (5) at least one intra-chromosome contact present in each autosome after filtering out reads with either end mapped to the ENCODE blacklist regions^58^ (Supplementary Fig. 1a).

### Genotype quality control and imputation in the Tilkka cohort

We genotyped the DNAs from the Tilkka participants using the Infinium Global Screening Array-24 (v.1) (Illumina). In our quality control, we used PLINK (v.1.9)^59^ to remove individuals with missingness of >2%; unmapped, strand-ambiguous and monomorphic single nucleotide polymorphisms; and variants with missingness of >2% and Hardy–Weinberg equilibrium *P* < 10^−6^. In addition, we imputed biological sex using the ‘–sex-check’ function in PLINK (v.1.9)^59^ and confirmed that they matched the reported sex for all individuals.

We used the HRC reference panel (v.r1.1 2016)^60^ to perform genotype imputation on the Michigan imputation server. Before imputation, we removed duplicate variants as well as variants with allele mismatch with the HRC reference panel, and we matched strand flips and allele switches to the panel before haplotype phasing using Eagle (v.2.4)^61^. To perform the genotype imputation, we used minimac4 (ref. ^62^) and performed quality control on the data by removing single nucleotide polymorphisms with an imputation score R^2^ < 0.3 and Hardy–Weinberg equilibrium *P* < 10^−6^.

### SnRNA-seq of human SAT in the Tilkka cohort

We performed SAT snRNA-seq experiments (Supplementary Note 2.1) on the snap-frozen SAT biopsies from the Tilkka participants. We measured the concentration and quality of nuclei, separately for each sample, using a Countess II FL Automated Cell Counter after staining with trypan blue and Hoechst dyes. To construct the libraries, we used the Single Cell 3′ Reagent Kit (v.3.1) (10× Genomics) and analyzed the quality of cDNA and gene expression using an Agilent Bioanalyzer. We sequenced the libraries from each participant together on an Illumina NovaSeq S4 with a target sequencing depth of 600 million read pairs. Processing of the SAT snRNA-seq data from the Tilkka cohort is described in Supplementary Note 2.2.

### Snm3C-seq data integration, clustering and annotation

We aligned the raw snm3C-seq reads against the GRCh38 human genome reference and represented the mCG profiles of each cell by 5 kb bins across autosomal chromosomes. In brief, per cell and for each 5 kb bin, we calculated a hypomethylation score. Bins that overlapped with the ENCODE blacklist regions were excluded from the clustering analysis. Next, we performed latent semantic indexing to obtain the mCG profile embedding (Supplementary Note 2.3). For the chromosome conformation modality, we imputed the contact matrix of each cell at 100 kb resolution using scHicluster^63^ (v.1.3.5) with pad = 1 and used singular value decomposition to project all intra-chromosome contacts between 100 kb and 10 Mb that land in autosomal chromosomes to a low-dimensional space (Supplementary Note 2.3).

We annotated the clusters de novo by leveraging the negative correlation between mCG and transcriptional activity^10,15,16^ and the known SAT marker genes, reported previously^64^. Specifically, we aligned the snm3C-seq data to GENCODE (v.33) annotations and calculated the average mCG fractions of the gene body (Supplementary Note 2.4).

We then tested for de novo gene body hypomethylation patterns across all protein-coding genes for each snm3C-seq cluster, defined using both the global mCG and chromatin conformation embeddings. To identify top cluster markers, we adapted criteria similar to those used for CH methylation in the brain^16^, applying them to mCG in SAT. In more detail, we required a hypomethylation difference of ≥0.15 in the average normalized fraction compared to other clusters, a Benjamini–Hochberg-adjusted Wilcoxon rank-sum test *P* value of ≤0.01 and an area under the receiver operating characteristic curve of ≥0.75 for distinguishing cells within the cluster from those outside it. Next, we leveraged the existing snRNA atlas of SAT cells for annotation^64^ (Supplementary Note 2.4).

For modality-specific analysis of snm3C-seq cells (that is, those independently identified within the global mCG or chromatin conformation profiles), we performed separate clustering, visualization and annotation similarly. The final annotations for each modality-specific cluster were determined by identifying the most common snm3C-seq cell-type labels among its constituent cells (Fig. 2b). We categorized the group of cells that clustered with adipocytes if using only the chromosome conformation information and with perivascular cells if using only the mCG profiles as the ‘transition’ cluster.

### SnRNA-seq data integration, clustering and annotation

We integrated all remaining high-quality droplets from all snRNA-seq data with reciprocal principal components analysis (rPCA) implemented in Seurat (v.4.3.0)^65^ and clustered integrated data with a standard Louvain algorithm, using parameters of the first 30 principal components and a resolution of 0.5. We annotated each cluster with its cell type using SingleR (v.1.8.1)^66^, using a previously published single-cell atlas of human SAT as a ref. ^64^.

### Co-embedding of snm3C-seq and snRNA-seq data

We aligned the snm3C-seq cells as the query and snRNA-seq cells as the refs. ^18,67^ under the canonical correlation analysis framework in Seurat (v.4.1.0)^19^. To capture the shared variance between modalities, we started by reversing the sign of the normalized gene body mCG fractions. In more detail, given that a decrease in gene body methylation is typically associated with increased transcriptional activity, we began by reversing the sign of the normalized gene body mCG fractions for all snm3C-seq nuclei to correct for the mismatch in directionality between the two modalities. As a result, in the adjusted mCG fraction matrix, cell-type marker genes show higher gene body methylation values in their respective cell types than other cell types, aligning with the patterns observed in the snRNA-seq data. These adjusted gene body methylation values serve as a proxy for the transcriptional activity of the snm3C-seq nuclei, which we then co-embedded with those from the snRNA-seq nuclei (Supplementary Note 2.5). Cells profiled by both technologies were merged on their imputed expression profiles, projected to low-dimensional space with PCA and visualized by uniform manifold approximation and projection (constructed on the top ten principal components). We evaluated the concordance score and the confusion matrix between each de novo snm3C-seq and the snRNA-seq cell-type cluster pair in the shared canonical correlation analysis co-embedding space (Supplementary Note 2.6). We also performed adipogenesis trajectory analysis using SAT snRNA-seq data (Supplementary Note 2.7).

### Cell-type-level SAT marker gene identification

We excluded the following genes from differential testing of the cell-type marker genes: genes that overlap with the ENCODE blacklisted regions; smaller genes (≤200 bp) mostly covered by other genes (overlap region of ≥90% of the gene length)^16^; and genes with a shallow coverage, constantly methylated or un-methylated, defined as those without ≥10 methylated or un-methylated counts in ≥10 cells belonging to the cell type under investigation. Cell-type-level differentially methylated genes were determined de novo by performing a Wilcoxon rank-sum test on the normalized gene body mCG fractions of the snm3C-seq cells in a one-vs-rest way. We retained genes that had a Benjamini–Hochberg adjusted *P* value of <0.05 and at the same time exhibited a hypomethylation difference of ≥0.1 in terms of the average normalized fraction compared to the other cell types^16^. For transcriptomics, we first filtered for the set of expressed genes in SAT, defined as those with ≥3 counts in ≥3 cells^68^. We used the ‘FindAllMarkers’ function in Seurat under default parameters except restricting to positive marker genes with ≥25% non-zero expression in either the tested cell type or the other ones^69–71^. Subsequently, we filtered out genes with Bonferroni-adjusted *P* ≥ 0.05. To obtain unique marker genes from both snm3C-seq and snRNA-seq, we removed genes identified as marker genes for more than one cell type.

### Pathway enrichment analyses for SAT cell-type marker genes

To identify cell-type-level biological processes and functional pathways enriched among the cell-type marker genes in mCG and gene expression modalities, we used the web-based tool WebGestalt 2024 (ref. ^72^), which identifies the overrepresentation of gene sets in Gene Ontology biological processes and KEGG pathways. For each SAT cell type, we used the unique cell-type marker genes as the input, with only the genes expressed within that cell type as the reference for the enrichment analysis. Biological processes and KEGG pathways with FDR < 0.05 were considered statistically significant.

### Cell-type-level methylation profile analysis

To obtain the cell-type-level mCG profiles, we aggregated the single-cell-level number of CG methylated counts and total coverage based on the snm3C-seq joint annotation and further merged reads mapped to adjacent CpGs in +/− strands. We then used MethylPy^20,21^, implemented in the ALLCools package, to detect genomic regions that display distinct mCG patterns across various cell types. DMSs on autosomes were tested across all eight annotated cell types using default parameters. For all DMSs, we assigned one of the three states per cell type—hypomethylated, neutral or hypermethylated—based on whether the fitted residual (that is, the normalized deviation away from the mean methylation level) fell below the 0.4, between the 0.4 and 0.6 or above the 0.6 quantile of its chromosome-wide background, respectively^16^. Nearby DMSs (within 250 bp) with Pearson correlations of >0.8 for the methylation fractions across the cell types were merged into DMRs. Differential methylation states were assigned to each DMR based on the average of those of the DMSs it encompasses. DMRs containing only one DMS or without any hypermethylation or hypomethylation state assignment, along with DMRs or DMSs overlapping ENCODE blacklist regions, were excluded from downstream analyses. We further evaluated cell-type preferential DMR patterns, adjusting for variability in statistical power owing to differences in coverage across cell types (Supplementary Note 2.8).

### Prediction of cell-type-level TF binding motifs

We performed TF binding motif enrichment analysis using the motif discovery tool HOMER (Hypergeometric Optimization of Motif EnRichment) (v.4.11.1)^22^. For each SAT main cell type, we used the hypomethylated regions as input data for motif enrichment analysis with the HOMER function ‘findMotifsGenome.pl’. TF binding motifs with *P* < 1 × 10^−12^ were considered statistically significant. Circular visualization of cell-type-level TF binding motif enrichment results was prepared using the circlize package (v.0.4.16)^73^ in R. We further validated cell-type-level TFs by confirming the overrepresentation of ChIP-seq peaks overlapping DMRs (Supplementary Note 2.9), using external data from ENCODE^23^.

### Cell-type-level compartment analysis

Based on the snm3C-seq joint annotation, we merged scHicluster imputed single-cell-level contact matrices at 100 kb resolution per chromosome to form the cell-type-level pseudobulk conformation profiles for the five most abundant cell types (adipocytes, ASPCs, endothelial, perivascular and myeloid cells) as well as a cell-type aggregated version (Supplementary Note 2.10). Cell-type-level pseudobulk conformation profiles were then normalized by the distance between the contacts and converted to correlation matrices by dcHiC (v.2.1)^46^. For all five cell types, we fitted PCA on the resulting matrices per chromosome and selected the appropriate leading principal component as the compartment scores (Supplementary Note 2.11). Finally, we tested for genomic bins that demonstrated large deviations from the cell-type average under a multivariate normal distribution measured by the Mahalanobis distance using the covariance matrix learned with outlier bins removed. Bins with FDR-corrected *P* < 0.1 were labeled as differentially conformed regions^46^. Empirically, we observed FDR < 0.02 when repeating the same analysis but only on a null set of cell-type-level contact maps, obtained by arbitrarily shuffling the annotation of the cells before merging to the pseudobulk level (that is, a scenario in which any differential compartment detected is false-positive by construction), indicating a conservative calibration of the testing result by dcHiC.

### Characterizing domains and chromatin loops in SAT

For interaction domains, we used scHiCluster (v.1.3.5)^63^ with pad =2 to impute the contact matrix of each cell per autosomal chromosome at a 25 kb resolution, restricted to contacts within 10 Mb. We detected domains for each cell using TopDom (v.0.0.2)^74^, calculated the insulation scores across all 25 kb genomic bins with a window size of ten bins using the imputed contact profiles and visualized with uniform manifold approximation and projection (Supplementary Note 2.12). Cell-type-level domain boundary probabilities were calculated as the fraction of cells with a detected domain boundary in a given 25 kb bin across all cells belonging to the specified cell type. Differential domain boundaries were evaluated per bin based on the following criteria^45^: a *z*-score-transformed chi-squared statistic of >1.960 (97.5^th^ percentile of standard normal distribution); differences between the maximum and minimum cell-type boundary probabilities of >0.05; detection as a local boundary peak (maximum); simultaneous detection as a local insulation score valley (minimum); and FDR < 0.001.

To analyze chromosomal looping, we used scHiCluster (v.1.3.5)^63^ with pad = 2, window_size = 30,000,000 and step_size = 10,000,000 to impute a contact matrix of each cell per autosomal chromosome at a 10 kb resolution, restricted to contacts within 10 Mb. Loop pixels were detected from cell-type pseudobulk imputed contact profiles based on enrichment relative to both its global and local backgrounds. We aggregated nearby loop pixels passing an empirical FDR of 0.1 to loop summits (Supplementary Note 2.12).

### Further analyses of methylation pathway genes

We assessed associations between *DNMT3A* and *TET1* SAT bulk expression and key CMD traits, including WHRadjBMI, BMI, serum triglyceride levels, Matsuda Index and fat mass (Supplementary Note 2.13). Using six-time-point preadipocyte differentiation data (Supplementary Note 2.14), we also analyzed longitudinal co-expression patterns of adipogenesis genes and their methylation regulators (Supplementary Notes 2.15 and 2.16).

### Partitioned cardiometabolic PRSs for DMRs and compartments

To assess the contributions of the SAT cell-type-level DMRs and cell-type-level compartments on the genetic risk for cardiometabolic traits, we constructed partitioned PRSs from variants landing in each set of cell-type-level DMRs and compartments for BMI, WHRadjBMI, CRP and MASLD in the UKB^30,31^, using a previously published imputed MASLD status^75^.

We first generated GWAS summary statistics for each trait with a 50% base group (*n* = 195,863) by applying a rank-based inverse-normal transform to each trait and used the linear-mixed model approach of BOLT-LMM (v.2.3.6)^76^, including age, age^2^, sex, the top 20 genetic principal components, testing center and genotyping array as covariates. Variants with a minor allele frequency of <1% and INFO < 0.8 were removed from the summary statistics. We then partitioned the remaining 50% into a 30% target and 20% validation groups for developing and applying the PRS model, respectively. Variants with a minor allele frequency of <1% and INFO < 0.8 were removed from the GWAS summary statistics, and the variants missing in >1% of subjects, with a minor allele frequency of <1% or violating Hardy–Weinberg equilibrium, as well as individuals with >1% missing genotypes or extreme heterozygosity, were removed from the target and validation genotype data^77^.

To compute the PRS for each outcome, we first generated independent marker sets by performing linkage disequilibrium clumping on all variants passing quality control in the genome using PLINK (v.1.9)^78^, with a linkage disequilibrium R^2^ threshold of 0.2 and a window size of 250 kb. We then used the 30% test set (*n* = 115,120) to empirically identify the optimal *P* value cut point at the genome-wide level. In brief, we applied the PLINK^78^ ‘–score’ functionality to separately compute aggregated scores from subsets of the genome-wide clumped variants passing a *P* value threshold from 5 × 10^−8^ to 0.5, using effect sizes and *P* values from the GWAS summary statistics. After identifying the best thresholding cutoff in the 30% test set (0.05 for WHRadjBMI and CRP, 0.3 for BMI and 0.2 for MASLD), we computed regional PRSs in the 20% validation set (*n* = 76,758), consisting of the clumped and thresholded variants landing within the DMRs or compartments. Variance explained (R^2^) by each PRS was calculated by adjusting each trait for age, age^2^, the top 20 genetic principal components, testing center, genotyping array and sex, applying a rank-based inverse-normal transform and then regressing the PRS on the adjusted trait. We assessed the statistical significance of the variance explained by each PRS by comparing it to those of 10,000 permuted PRSs constructed from randomly selected variant sets with matching size and genomic background (that is, genome-wide clumped and thresholded). We also assessed overrepresentation of GWAS variants (Supplementary Note 2.17), their *cis*-expression quantitative trait locus effects and relevance to CMDs (Supplementary Note 2.18).

### Statistics and reproducibility

No statistical method was used to predetermine the sample size of the snm3C-seq cohort; however, our sample sizes and number of cells profiled are similar to those reported in previous snm3C-seq publications^12^. No samples were excluded from the Tilkka cohort. In the UKB and METSIM cohorts, related individuals were excluded. As this is an observational study, no randomization was performed. Data collection and analysis were performed blind to the conditions of the experiments. Results obtained from SAT snm3C-seq and snRNA-seq were not replicated owing to the unique nature of these datasets. Results reported from the UKB GWAS analyses were not replicated because of the unprecedentedly large sample size of the cohort. Correlations observed with the METSIM bulk RNA-seq data were not replicated because of the unique refined phenotype profiles of the METSIM cohort. Statistical significance was assessed using empirical permutation or non-parametric tests when possible. Assumed data distributions for other analyses (binomial, hypergeometric, multivariate normal) were not formally tested. Owing to the large sample size (for example, genomic bins, ChIP-seq peaks), individual data points were not visualized.

### Reporting summary

Further information on research design is available in the Nature Portfolio Reporting Summary linked to this article.

## Online content

Any methods, additional references, Nature Portfolio reporting summaries, source data, extended data, supplementary information, acknowledgements, peer review information; details of author contributions and competing interests; and statements of data and code availability are available at 10.1038/s41588-025-02300-4.

## Supplementary information

Supplementary Information Supplementary Notes, Supplementary Figs. 1–7 and References.

Reporting Summary

Supplementary Tables 1–14 Supplementary Tables 1–14.

## Data Availability

The data that support the findings in this manuscript are available from the UKB. However, restrictions apply to the availability of these data, which were used in this study under UKB application number 33934. UKB data are available for bona fide researchers through the application process at https://www.ukbiobank.ac.uk/learn-more-about-uk-biobank/contact-us. The snm3C-seq and snRNA-seq data from the Tilkka cohort are available in the Gene Expression Omnibus (GEO) under accession number GSE297267, along with the epigenomic annotations of SAT DMRs, compartments, domains and loops characterized in this study. The GRCh38 reference genome is available through the UCSC genome browser (https://hgdownload.soe.ucsc.edu/goldenPath/hg38/bigZips)^79^. The bulk RNA-seq data from the primary human preadipocyte differentiation experiment were previously made available in GEO under accession number GSE249195. The METSIM SAT bulk RNA-seq data^55^ are available in GEO under accession number GSE135134. Bulk SAT *cis*-expression quantitative trait locus variants are available from the GTEx v10 cohort^80^. ChIP-seq datasets are publicly available on the ENCODE portal with the following accession numbers: ENCSR000BGY (https://www.encodeproject.org/experiments/ENCSR000BGY), ENCSR177VFS (https://www.encodeproject.org/experiments/ENCSR177VFS) and ENCSR490LWA (https://www.encodeproject.org/experiments/ENCSR490LWA). Source data files and GWAS summary statistics from this study are available on Zenodo (https://zenodo.org/records/15318595)^81^.

## Code availability

All packages and software used in this study were from their publicly available sources, as outlined in the Methods. No custom code was generated.
