# Supplementary material for: Single-cell DNA methylome and 3D genome atlas of human subcutaneous adipose tissue
Source: Nat Genet. 2025 Aug 20;57(9):2238–49. doi: 10.1038/s41588-025-02300-4 (PMC12373012; doi:10.1038/s41588-025-02300-4)
Supplement: Supplementary file 1 — Supplementary Notes, Supplementary Figs. 1–7 and References. [file 41588_2025_2300_MOESM1_ESM.pdf]

nature genetics

Supplementary information https://doi.org/10.1038/s41588-025-02300-4

Single-cell DNA methylome and 3D genome atlas of human subcutaneous adipose tissue

In the format provided by the authors and unedited

SUPPLEMENTARY NOTE

Contents

1. Supplementary Results

1.1. Overview of the study design

1.2. Quality control of snm3C-seq

1.3. Quality control of snRNA-seq

1.4. Biological interpretation of the transitional cell-type

1.5. Unique marker gene comparisons: mCG vs. gene expression

1.6. Adipocyte marker enriched pathways and biological processes

1.7. Adipocyte marker genes that encode transcription factors

1.8. Cell-type level transcription factor binding motifs

1.9. Short- and long-range interactions across SAT cell-types

1.10. Differences in cell-type level chromosome compartments

1.11. ASPC compartment changes on gene regulation

1.12. Further investigation of *TET1* and *DNMT3A*

1.13. Temporal co-expression of *TET1* with known adipogenesis genes

1.14. Enrichment of the partitioned PRSs and GWAS variants

2. Supplementary Methods

2.1. Nuclei isolation and snRNA-seq of human SAT

2.2. Processing of the SAT snRNA-seq data from the Tilkka cohort

2.3. Modality-specific embedding of snm3C-seq data

2.4. Evaluating gene-body mCG profiles

2.5. Co-embedding using CCA analysis

2.6. Evaluating cross-modality annotation concordance

2.7. Adipogenesis trajectory analysis in SAT snRNA-seq

2.8. Statistical testing of cell-type preferential DMR patterns

2.9. Validation of cell-type level TFs

2.10. QC of 100-kb genomic bins

2.11. Principal component selection for compartment scores

2.12. UMAP visualization of interaction domains and loops

2.13. SAT bulk associations between *DNMT3A* and *TET1* and CMD traits

2.14. Human primary preadipocyte differentiation experiment

2.15. Longitudinal differential expression across adipogenesis

2.16. Longitudinal trajectories of co-expressed genes

2.17. Overrepresentation of GWAS variants in DMRs and compartments

2.18. Transcriptomic impact of GWAS variants in cell-type DMRs

Supplementary Figures

References

1. Supplementary Results

1.1. Overview of the study design

Epigenomic landscape of human subcutaneous adipose tissue (SAT) is unknown at the cell-type level. To address this knowledge gap, we used snm3C-seq (n=5 samples) and snRNA-seq (n=8 samples) technologies on nuclei isolated from SAT biopsies from Finnish females (see Methods) (Fig. 1a) to generate cell-type level DNA methylation, chromatin conformation, and gene expression profiles in SAT. After performing careful quality control (QC) in each modality, we verified the high concordance of cell-type annotations derived from CG methylation (mCG) and interaction modality as well as between mCG and gene expression. We then conducted analysis of differentially methylated regions (DMRs) to find cell-type level differences in DNA methylation patterns in SAT (Fig. 1b). To elucidate chromatin conformation dynamics in SAT cell-types, we systematically searched for cell-type level patterns in terms of the global contact distance distribution, as well as 3D genome features at various resolution (i.e., compartments, domains, and loops) (Fig. 1c). We next utilized cell-type level SAT snRNA-seq data (Fig. 1d) to investigate whether methylation pathway genes contribute to the discovered differences in DNA methylation patterns in SAT cell-types and cluster with adipogenesis pathway genes (Fig. 1e). We also identified cell-type level TF binding motifs associated with hypo-methylated regions of SAT cell-types (Fig. 1f). Finally, to understand how these cell-type level epigenomic differences relate to the key cardiometabolic phenotypes relevant to SAT, we examined whether variants in cell-type level DMRs and compartments contribute significantly to the polygenic risk of obesity and related cardiometabolic traits (Fig. 1g).

1.2. Quality control of snm3C-seq

A total of 6,652 nuclei passed our QC, with each cell having on average of 2,215,681 non-clonal methylation reads, 236,849 chromatin contacts, and uniform representation across the human genome (Supplementary Fig. 1a,b), covering a mean (±SD) of 29,251 (±1,953) genes and of 97.3% (±1.7) non-overlapping 100-kb bins, similar to previous human studies in solid tissues[1,2]. We independently identified 7 main cell-types using the global mCG of non-overlapping 5-kb bins and the intrachromosomal contacts among non-overlapping 100-kb bins (Fig. 2a and Supplementary Figs. 2, 3). The annotated cell-types and their mean proportions (±SD) across the 5 samples are as follows: adipocytes, 16.3% (±7.3); adipose stem and progenitor cells (ASPCs), 28.8% (±6.8); perivascular, 7.1% (±1.3); endothelial, 17.0% (±6.2); myeloid, 22.0% (±8.1); lymphoid, 5.4% (±4.7); and mast cells, 2.7% (±1.0).

1.3. Quality control of snRNA-seq

First, we applied snRNA-seq on nuclei isolated from the same 5 SAT samples and 3 additional SAT samples from the Tilkka cohort to obtain 29,423 SAT nuclei and their single-cell level expression profiles (see Methods). Each snRNA-seq nucleus that passed QC has a mean (±SD) of 4,741 (±4,334) counts, 2,001 (±1,154) number of expressed genes, and 0.2% (±0.6) mitochondrial expression (Supplementary Fig. 1c), on par with a previous snRNA-seq study performed on human SAT[3] with a mean (±SD) of 4,771 (±5,723) counts, 2,001 (±1,216) expressed genes, and 1.83% (±2.02) mitochondria expression. We annotated the snRNA-seq nuclei at the matching cell-type resolution (Extended Data Fig. 1a), resulting in a mean proportion (±SD) of 43.5% (±14.7) adipocytes, 22.5% (±7.5) ASPCs, 4.3% (±3.4) perivascular, 9.3% (±6.9) endothelial, 16.1% (±7.2) myeloid, 2.9% (±2.7) lymphoid, and 1.3% (±1.2) mast cells.

1.4. Biological interpretation of the transitional cell-type

Noteworthy, depending on the number of canonical correlation vectors (CCVs) used to construct the shared low dimensional space, the transition cell-type cluster was co-embedded close to either the adipocytes profiled by the snRNA-seq, or the perivascular cells (i.e., its *de novo* global mCG annotation) (Fig. 2b,d,e, Extended Data Fig. 1c,d, and Supplementary Fig. 5). Analysis of the gene body mCG levels further revealed that it simultaneously shows diminished methylation levels on both adipocyte and perivascular marker genes, with overlaps of 23.1% and 44.5%, respectively, while showing minimal demethylation on other cell-type marker genes (below 2.5%) (Fig. 2c and Extended Data Fig. 2g).

Our latent time analysis using snRNA-seq splicing data (Supplementary Fig. 6a) further indicated that the genes involved in the regular adipocyte differentiation (i.e., adipogenesis) route from ASPCs to adipocytes significantly overlap with the transition cell-type marker genes (OR=4.5, $P=2.7\times10^{-13}$, see Methods, Supplementary Fig. 6b,c). In addition, when restricted to the relevant cell-types, the transition cell-type cluster could also be discerned from the low dimensional projection of the genome-wide 5-kb bin mCG profiles, i.e., in the absence of chromatin conformation information (Extended Data Fig. 2h). The above-mentioned mCG properties at the individual transcriptomics level and the global projection underscore the biological validity of the transition cell-type.

During the differentiation of other tissues, the establishment of global chromatin 3D structure has previously been shown to precede the formation of methylation signatures[4]. In other words, the observed asynchrony between the mCG and conformation profiles suggests that the transitional cell-type cluster is undergoing active differentiation from perivascular cells to mature adipocytes,

in line with recent studies that discovered perivascular adipocyte progenitors in mice and humans[5–7].

The dynamic and asynchronous nature of SAT cell-types across modalities is exemplified by the identification of the transition cell population. Current evidence from perivascular adipose tissue in both mice and humans indicates that the perivascular adipocyte progenitor cells undergo adipocyte differentiation via induction of a thermogenic gene program[5]. Studies on rodent models have shown that *Ebf2*, a TF gene we also found to be hypo-methylated in the transition cell-type, is selectively expressed in mouse precursor cells of brown or beige fat[8] and regulates thermogenic gene programming in mouse adipocytes[5,9]. This suggests that the observed transition cell-type represents the brown fat progenitor cells, which further differentiate into adipocytes in response to selective epigenomic and likely also environmentally driven changes.

1.5. Unique marker gene comparisons: mCG vs. gene expression

We first searched for differences in unique marker genes at the cell-type level between the gene body mCG and gene expression modalities (Supplementary Tables 1-2) and found both modality-specific and -shared marker genes (Extended Data Fig. 3a). We observed that majority of the cell-type level marker genes were identified as modality-specific. For instance, 77 adipocyte marker genes are present in both modalities, while 286 are unique to gene body mCG and 738 are unique to gene expression.

1.6. Adipocyte marker enriched pathways and biological processes

For example, the PPAR signaling pathway, a well-known adipose tissue pathway, is significantly enriched among the adipocyte marker genes in both modalities at a false discovery rate (FDR)<0.05. We also identified several other shared biological processes, including fat cell differentiation, enriched among the adipocyte marker genes. Enrichment of these shared biological processes and functional pathways between the two modalities suggests that both methylation and gene expression play roles in regulating cell-type level molecular mechanisms.

1.7. Adipocyte marker genes that encode transcription factors

We also investigated whether any of the adipocyte marker genes in gene body mCG and gene expression modalities are TFs[10], and whether they have previously been characterized across the SAT cell-types. Among the shared adipocyte marker genes across the two modalities, we found both known and less known TFs in SAT, including *TCF7L2*, *EBF1*, *SREBF1*, *KLF15*, and *STAT5A* (Extended Data Fig. 3e). Additionally, we identified unique gene body mCG marker genes in adipocytes that encode TFs. While some of these TFs, such as RXRA, are well-characterized in adipose tissue[11], the majority of them (e.g., PIN1, ZNF740, BRF2, NFIL3, SLC2A4RG) have not previously been extensively investigated in the SAT cell-types (Extended Data Fig. 3f), thus providing additional less known TFs for further functional investigation in SAT.

1.8. Cell-type level transcription factor binding motifs

We first identified significantly ($P<1\times10^{-12}$) enriched TF binding motifs for each SAT cell-type using HOMER[12]. Hypo-methylated region -associated TFs and their corresponding enrichment ratios and P values are listed in Supplementary Table 4. Next, we searched for cell-type level TFs present in one cell-type and absent in others (Fig. 3c and Extended Data Fig. 4). Among the cell-

type level TFs, the hypo-methylated regions in adipocytes are enriched for Twist family basic helix-loop-helix type transcription factor 2 (Twist2), homeobox A9 (HOXA9), and CCAAT enhancer binding protein delta (CEBPD); ASPCs for Twist family basic helix-loop-helix type transcription factor 1 (TWIST1), SMAD family member 3 (Smad3), and Jun proto-oncogene (JUN); and myeloid cells for CCAAT enhancer binding protein epsilon (CEBPE), Activating transcription factor 4 (ATF4), and Interferon regulatory factor 4 (IRF4). Our findings suggest that these TFs might either bind to the DNA in a cell-type-specific manner or regulate cell-type level differential methylation patterns.

Among the adipocyte-specific TFs, TWIST2 was identified as the top hit. A recent mice study reported that Twist2, a basic helix-loop-helix (bHLH) type TF, plays an essential role in lipid uptake and adipogenesis[13]. We also found an ASPC-specific TF, SMAD3, which acts as a downstream transcriptional transducer in the activin signaling pathway[14]. This pathway is well-studied for its role in the proliferation, differentiation, and function of preadipocytes[14,15]. Myeloid-specific TFs, CEBPE and ATF4, are known to regulate the expression of myeloid-specific genes[16]. These results endorse the possibility that both cell-type level hypo-methylation and TFs enriched in these hypo-methylated regions contribute to the regulation of gene expression in SAT in a cell-type-specific manner.

1.9. Short- and long-range interactions across SAT cell-types

The median proportion of the short-range interactions in adipocytes and the transition cell-type is 36.5%, whereas the median for others is 29.9%. Similarly, for long-range interactions, the median proportion is 26.0% both in adipocytes and the transitional cell-type, compared to 32.2% in others.

1.10. Differences in cell-type level chromosome compartments

Measuring the number of consecutive 100-kb bins within segments that exhibit consistent A or B compartment annotations, the median values are 15, 15, and 13 for adipocytes, ASPCs, and perivascular cells, respectively, whereas the medians are only 10 and 8 for myeloid and endothelial cells ($-\log_{10}P>25$, one-tailed Wilcoxon rank-sum test) (Extended Data Fig. 6d).

Across all SAT cell-types, the widespread differences are reflected in the observed differential conformations detected in 44.3% of the 100-kb bins. When focusing on compartment flips relative to adipocytes, a marked higher proportions of differential 100-kb bins categorized as adipocyte B compartments correspond to the A compartments of endothelial and myeloid cells (41.4% and 41.6%), in contrast to those of APSC and perivascular cells (28.0% and 33.8%, respectively; Extended Data Fig. 6f,g).

1.11. ASPC compartment changes on gene regulation

COL1A2 and *LAMA2* are highly expressed in ASPCs and are essential to the early stage of adipogenesis[17], responsible for the extracellular matrix formation[18,19]. As the adipocytes mature, the regions harboring them flip from ASPC A to adipocyte B compartments, repressing the transcriptomic activity and halting the cell proliferation and tissue remodeling.

1.12. Further investigation of *TET1* and *DNMT3A*

We further investigated *TET1* and *DNMT3A* for associations with five key obesity-related cardiometabolic phenotypes, including body mass index (BMI), Matsuda index (MI), serum

triglyceride levels (TGs), fat mass, and waist-hip-ratio adjusted for BMI (WHRadjBMI) in a SAT bulk RNA-seq dataset (n=335)[20–22] from the Finnish METabolic Syndrome In Men (METSIM) cohort[23]. We found that *DNMT3A* expression is significantly positively associated with TGs (FDR=0.044) and negatively associated with insulin sensitivity, measured by MI (FDR=0.032) (Supplementary Table 8), thus connecting high SAT bulk expression of this gene to an adverse direction of the obesogenic cardiometabolic traits, TGs and MI. In our single-cell level analysis (Fig. 5b), we in turn identified *DNMT3A* to be predominantly expressed in the SAT myeloid cells, the key cell-type for the obesity-induced adverse CMD outcomes and low-grade inflammation in SAT[24]. Conversely, we found that *TET1* SAT bulk expression is significantly negatively correlated with fat mass (FDR=0.03), and positively associated with MI (FDR=0.032) (Supplementary Table 8), thus linking the high *TET1* SAT bulk expression to healthy directions of the two CMD traits. At the single-cell level, we in turn observed that *TET1* is predominantly expressed in the SAT adipocytes (Fig. 5b) and *DNMT3A* in myeloid cells, with minimal expression in adipocytes (Fig. 5b). We note that none of the previous snRNA-seq studies specifically examined the role of *DNMT3A* in human adipose tissue cell-types - adipocytes, ASPCs, perivascular, endothelial, myeloid, lymphoid, and mast cells.

1.13. Temporal co-expression of *TET1* with known adipogenesis genes

As temporal expression and co-expression patterns across human adipogenesis may relate to differential methylation between ASPCs and adipocytes, we examined longitudinal expression of 124 known adipogenesis pathway genes along with 5 demethylase and methylase genes (*UHRF1*, *TET1*, *TET2*, *TET3*, and *TDG*) across 6 time points of differentiation of human SAT primary preadipocytes (i.e., adipogenesis) (see Methods). We first observed that as expected, 121 of the

tested known adipogenesis genes were longitudinally differentially expressed (DE) during SAT differentiation (adjusted $P<0.05$). To assess how the temporal co-expression patterns during adipogenesis relate to the expression of these demethylases and methylases, we clustered these genes using DPGP[25] into 14 distinct clusters of longitudinally co-expressed genes (Supplementary Table 9). Notably, *TET1*, which we showed to be preferentially expressed in adipocytes when compared to the other methylase and demethylase genes (Fig. 5b and Extended Data Fig. 9a,b), clustered with known adipogenesis TFs and functionally important SAT genes, including *ADIPOQ*, *PLIN1*, *CEBPA*, and *LPL*. All exhibit significant demethylation and increased expression toward the end of adipogenesis (Fig. 5e,f). This suggests that *TET1* may function as a potentially important demethylation regulator of genes involved in adipogenesis.

1.14. Enrichment of the partitioned PRSs and GWAS variants

For DMRs, we observed that 6 of the WHRadjBMI PRSs, constructed from variants in adipocyte, ASPC, and endothelial hypo-methylated, and perivascular, endothelial, and myeloid hyper-methylated regions, have significantly higher incremental variance explained than expected ($P_{perm10,000}<0.05$) (Fig. 6b and Supplementary Table 11). Moreover, 17.4%, 19.5%, 33.3%, 14.3%, 21.4%, and 17.8% of the respective nearby SAT *cis*-expression quantitative trait locus (*cis*-eQTL) target genes (eGenes) (see Methods), regulated by the *cis*-eQTL variants residing in the cell-type level DMRs, demonstrate enrichment of strong genetic contributions to cardiometabolic traits[26] (HuGE scores≥30; $P_{perm10,000}\leq0.0084$; Supplementary Table 12; see Methods). No PRS enrichments were observed for BMI or MASLD. We also observed significant enrichment of the WHRadjBMI GWAS variants in the adipocyte and myeloid hypo-methylated regions, as well as the ASPC and perivascular hyper-methylated regions (Fig. 6c and Supplementary Table 14). Their

adjacent, next-by genes similarly show a strong genetic contribution to obesity-related outcomes, with 61.8%, 36.3%, 38.5%, and 70.0%, respectively, having HuGE scores≥30. The binding sites of our *de novo* TFs identified in the myeloid hypo-methylated regions (i.e., IRF4, MEF2B, and CEBPG; Fig 3c, d), also exhibit a higher than expected chance of harboring such WHRadjBMI GWAS variants ($-\log_{10}P>2.42$; one-tailed hypergeometric test). No GWAS enrichments were seen for any of the cell-type level B compartments.

2. Supplementary Methods

2.1. Nuclei isolation and snRNA-seq of human SAT

To maximize the samples size of the snRNA-seq data in the Tilkka cohort, we performed the joint snRNA- and snATAC-seq experiment on the subset of 5 SAT biopsies and included the snRNA-seq data in this study. Briefly, we combined 300 mg of the 5 SAT biopsies into a gentleMACS C tube (Miltenyi Biotec) containing 3 ml of chilled 0.1X lysis, including 10 mM Tris-HCl, 10 mM NaCl, 3 mM MgCl2, 0.1% Tween-20, 0.1% IGEPAL CA-630, 0.01% Digitonin, 1% BSA, 1 mM DTT, and 1 U/µL RNase inhibitor. We next dissociated the tissues by placing the gentleMACS C tube on the gentleMACS Dissociator (Miltenyi Biotec) and running the '4C_nuclei_1' program. The tissues were incubated in the lysis buffer for a total of 15 minutes including the time on the dissociator. After the incubation period, we added 3 ml of chilled wash buffer, containing 10 mM Tris-HCl, 10 mM NaCl, 3 mM MgCl2, 1% BSA, 0.1% Tween-20, 1 mM DTT, and 1 U/µL RNase inhibitor, to the lysate and filtered the lysate mixture through a 70 µm MACS strainer, followed by a 30 µm MACS strainer. Next, the nuclei were centrifuged at 300g for 5 minutes at 4°C and the supernatant was removed without disrupting the nuclei pellets. We then resuspended the nuclei pellet in 3 ml of chilled wash buffer and passed through a 30 µm MACS strainer. The final concentration and quality of nuclei were measured using the Countess II FL Automated Cell Counter after staining with trypan blue and Hoechst dyes and the snRNA-seq library was constructed using the Single Cell Multiome ATAC + Gene Expression Reagent Kit (10x Genomics). We used the Agilent Bioanalyzer to assess the quality of cDNA and sequenced the library on an Illumina NovaSeq SP with a target sequencing depth of 400 million reads.

2.2. Processing of the SAT snRNA-seq data from the Tilkka cohort

First, we aligned the raw snRNA-seq data from all experiments against the GRCh38 human genome reference and GENCODE v42[27] annotations with STAR v2.7.10b[28]. We utilized the '--soloFeatures GeneFull' option to account for full pre-mRNA transcripts. Then the quality of the raw and mapped snRNA-seq data were evaluated using FastQC v0.11.9. To remove empty droplets as well as nuclei with high levels of ambient RNA, we ran DIEM v2.4.0[29] with initialization parameters 1) UMI cutoffs ranging from 100 to 1000 to define debris, and 2) k=50 for the initialization step with k-means clustering, along with all other default parameters. We applied the sample specific UMI cutoffs in the initialization step to account for differences in sequencing depth between samples. We conducted the following filtering criteria[30]: We removed clusters with low average UMIs, low average number of unique genes detected (nFeatures), high percentage of mitochondrial mapped reads (%mito), and high number of mitochondrial and ribosomal genes as top expressed features. Accordingly, droplets with nFeatures≤200, UMI≤500, %mito≥10, and spliced read fraction≥90% were removed using Seurat v4.3.0[31]. Next, we used Seurat v4.3.0[31] to log-normalize gene counts employing the 'NormalizeData' function; identify top 2,000 variable genes using the 'FindVariableFeatures' function; scale the gene counts to mean 0 and unit variance using the 'ScaleData' function; perform principal component analysis (PCA) using the 'RunPCA' function; and cluster the nuclei with a standard Louvain algorithm, using parameters of the first 30 PCs, and a resolution of 0.5, respectively.

To remove reads from ambient RNA molecules, we ran DecontX[32] from celda R package v1.14.2 with the removed low-quality nuclei as the background and the Seurat cluster assignment as the 'z'. We then removed nuclei with nFeatures≤200, UMI≤500, UMI≥30,000, and %mito≥10 based

on the remaining reads. For the multiplexed snRNA-seq data of 5 SAT biopsies from the joint snRNA- and snATAC-seq experiment, we ran demuxlet v2 from the popscle software tool[33] to identify the originating individual of each nucleus. Next, DoubletFinder v2.0.3[34] was employed to remove predicted doublets. Since DoubletFinder requires a predicted number of doublets as input, we used a pN-pK parameter sweep, as recommended[34], to select pN=0.25 and the most optimal pK value that maximizes the mean-variant normalized coefficient.

2.3. Modality-specific embedding of snm3C-seq data

Briefly, per cell and for each 5-kb bin, we calculated a hypo-methylation score (i.e., the *P* value of observing fewer methylated reads under a binomial distribution with the expected probability of a methylated read set to the global mCG rate of the cell, and the number of trials set to the coverage of the 5-kb bin). We next binarized the score matrix by converting nominally significant entries (i.e., P value<0.05) to 1 and the rest to 0[35]. Bins that overlapped with the ENCODE blacklist regions[36] were excluded from the clustering analysis. Next, we performed latent semantic indexing (LSI) on the term-frequency, inverse-log-document-frequency transformed matrix, implemented in the ALLCools package[37] (v.1.0.23) to obtain the mCG profile embedding, and then further omitted the first dimension due to its high correlation with sequencing depth.

To remove the sample level batch effect, we applied Harmony[38] (v.0.0.9) on the snm3C-seq joint embedding (i.e., the concatenation of the top 10 dimensions from both modalities). The resulting matrix was used for k-NN graph construction (k=25), Leiden consensus clustering, and uniform manifold approximation and projection (UMAP) visualization.

2.4. Evaluating gene-body mCG profiles

We normalized the fractions per cell by first taking the posterior of the mCG probability of each gene with a Beta distribution prior, representing the genome-wide mCG rate of the cell, before scaling by the inverse of it[37]. Thus, hypo-methylated SAT marker genes, characterized by normalized scores notably lower than the genome-wide average of 1, suggest strong expression patterns.

We overlapped our top (up to 50) cluster markers with the marker genes of relevant cell-types from the adipose atlas of Emont et al.[3], using the overlap fraction to assign an unambiguous annotation to each de novo cluster (Supplementary Figs. 2, 3). To align with the annotation resolution used in this study, we combined SMC and pericytes from Emont et al.[3] into a single "perivascular cells" category, while macrophages, dendritic cells, and monocytes were grouped together as "myeloid cells."

2.5. Co-embedding using CCA analysis

We restricted our analyses to the set of genes originally selected for integrating the RNA datasets, while also requiring >5 mapped reads across the snm3C-seq cells. Before applying CCA, we standardized both the log-normalized RNA-seq expression counts and the reversed normalized gene body mCG fractions, ensuring that for each feature, the mean across cells in every modality is 0 and the standard deviation is 1. The empirical distribution across cells profiled by both modalities is shown in Supplementary Fig. 4. The standardized mCG values typically follow a smoother trend, while the transcriptomic data often exhibit a bimodal distribution or a left-skewed distribution with a long tail, indicating expression in only a subset of cells. While the CCVs project

the two datasets into a correlated low-dimensional space, global differences in scale (e.g., when aligning fundamentally different data modalities) can still preclude comparing CCVs across datasets. To alleviate this risk, Seurat internally performs an additional step of L2-normalization on the CCA cell embeddings after dimensional reduction. Transfer anchors were identified within the top 30 L2-normalized CCVs as the top 5 mutual nearest neighbors. We further filtered and weighted the anchors by distances in the snm3C-seq joint embedding to impute RNA-based annotations and expression profiles of the snm3C-seq cells. ARI was used to evaluate the concordance between the *de novo* annotation and the imputed RNA-based annotation.

2.6. Evaluating cross-modality annotation concordance

For a given *de novo* snm3C-seq and the snRNA-seq annotated cell-type cluster pair, we defined the overlap score as the sum, across all clusters in the shared CCA co-embedding space, of the minimum proportion of cells in each modality-specific cluster that overlapped with a co-embedding cluster. Thus, the overlap score ranges from 0 to 1, where 0 indicates a complete separation and 1 indicates a perfect co-localization of modality-specific cells within the same co-embedding cluster. We normalized the multi-class confusion matrix per row by the number of cells to derive the confusion fractions. The confusion matrix was calculated by comparing the *de novo* annotations of the snm3C-seq cells with their intermediate imputed cell-type labels, which were determined through weighted votes from transfer anchors using snRNA-seq as reference.

2.7. Adipogenesis trajectory analysis in SAT snRNA-seq

We separately integrated a subset of the SAT snRNA-seq data, focusing on cells annotated as adipocytes and ASPCs, to compute the first order moments from the nearest neighbor graph

constructed from the integrated PCs. Genes involved in building the trajectory were limited to the top 2000 variable genes with ≥20 unspliced and spliced counts. We used the dynamical model from scVelo[39] v0.3.2 to obtain the latent time estimation per cell, calculated on both the unspliced and spliced counts for genes with maximum likelihood>0.1 (Supplementary Fig. 6). To identify genes that were differentially ranked across adipogenesis subclusters (i.e., adipogenesis rate trajectory genes), we performed a Welch's t-test using the "rank_velocity_genes" function with genes that passed spearman correlation of 0.3 between unspliced and spliced data from scVelo[39]. Finally, we conducted a gene overlap analysis using the Fisher's exact test from GeneOverlap v1.36.0 between the adipogenesis rate trajectory genes and transition cell-type hypo-methylated marker genes. The background genome set included all genes expressed in the SAT snRNA-seq dataset.

2.8. Statistical testing of cell-type preferential DMR patterns

Additionally, we tested whether DMRs showcase genome-wide cell-type preferential differential methylation states under a regression framework. Specifically, for every differential state (hyper- or hypo-methylation), we fitted the following regression model across all cell-types and all autosomal chromosomes. The response variable, the cell-type level fraction of the DMRs with the desired methylation state on a chromosome, was modeled by several independent variables, including the log normalized number of cells belonging to the corresponding cell-type and a one-hot encoded indicator for all cell-types in SAT. This approach allowed us to quantify the cell-type level contribution to the methylation state fraction while also calibrating for the inherent fraction differences induced by the varying statistical power arising from the differences in the coverage among cell-types. The stratification to hyper- and hypo-methylation states naturally suggests a

directionality in the test. Thus, we report the $\log_{10}P$ values derived from one-tailed t-tests, evaluating the probability of observing a larger positive contribution on the cell-type indicator variable under the null.

2.9. Validation of cell-type level TFs

To validate the cell-type level TFs, we next utilized external ChIP-seq data from ENCODE[40]. We overlapped the ChIP-seq peaks, passing a 0.05 FDR threshold from the original studies[41–43], with cell-type level hypo-methylated regions. The percentage was calculated as the fraction of DMRs overlapping one or more peaks, relative to the total number of hypo-methylated regions annotated for the cell-type. To construct the null distribution with matching genomic context and size, we generated 1,000 sets of permuted DMR annotations. For each set, we shuffled the genomic locations of the DMRs and re-assigned the original DMR state annotations to the shuffled positions. Empirical *P* values were calculated based on the frequency at which the percentage of the permuted DMRs overlapping peaks exceeded the observed one. We used a one-tailed hypergeometric test to calculate the theoretical *P* value for observing even greater overlap.

2.10. QC of 100-kb genomic bins

For each chromosome independently, genomic bins in the cell-type aggregated contact map with abnormal coverage, defined as the total number of interactions between itself and all other bins, were removed from the compartment analysis. Specifically, we kept bins with a coverage <99th percentile and above twice the 50th percentile minus the 99th percentile. This filtration typically removes poorly mapped regions like telomere, centromere, and blacklisted regions[1].

2.11. Principal component selection for compartment scores

The dcHiC tool heuristically selected the PC that maximized the absolute correlation with TSS and CpG density as the compartment scores and, if needed, flipped its sign to ensure that regions with positive scores corresponded to more active (A) compartments. We visually inspected the compartment scores to verify that they indeed captured the plaid pattern instead of the chromosome arms. Compartment scores from all 5 cell-types were then quantile normalized.

2.12. UMAP visualization of interaction domains and loops

We then projected the domain boundaries with LSI and insulation scores with PCA into low-dimensional space. Similar to snm3C-seq joint annotation, we applied Harmony v.0.0.9[38] to correct for batch effects and visualized the top 10 low-dimensional embeddings with UMAP.

To create cell-level embeddings based on looping features, we first gathered all identified loop pixels and built a binary cell-by-loop matrix, where each entry indicates whether at least one contact was detected in the cell at the corresponding loop pixel[1]. We used LSI to project the cell-by-loop matrix, Harmony to correct for batch effect, and UMAP to visualize the top 10 low-dimensional embeddings.

2.13. SAT bulk associations between *DNMT3A* and *TET1* and CMD traits

Leveraging the SAT samples processed for bulk RNA-seq (n=335)[20–22] from the METSIM cohort[23], we assessed associations between *DNMT3A* and *TET1* SAT bulk expression and five key cardiometabolic traits: WHRadjBMI, BMI, TGs, MI, and fat mass. SAT RNA-seq reads were mapped and aligned using the same pipeline as previously[20–22]. We next normalized the raw

expression matrix using trimmed mean of M-values (TMM) implemented in edgeR v3.40.2 and applied limma-voom v3.54.2[44]. We conducted the DE analyses while controlling for the SAT cell-type proportions, estimated in our previous study[21]. In addition, we controlled for the following technical factors: RNA Integrity Number (RIN), batch, percentage of uniquely mapped reads, percentage of reads mapped to mitochondrial genes, the median 3′ bias of the 1,000 most highly expressed transcripts, and the percentage of aligned bases mapped to intronic regions. For each cardiometabolic trait, we applied BH procedure to adjust the raw *P* values for testing multiple genes. Statistical significance was determined at FDR<0.05.

2.14. Human primary preadipocyte differentiation experiment

We previously cultured cryopreserved human primary SAT preadipocytes (Zen-Bio catalog # SP-F-2, lot L120116E) for adipogenesis (14-day preadipocyte differentiation) and conducted RNA-seq across 6 time points: 0d, 1d, 2d, 4d, 7d, and 14d[21]. Briefly, for each time point and modality, we plated cells at confluency to create 4 isogenic replicates. Libraries for RNA-seq were prepared using the Illumina TruSeq Stranded mRNA kit and sequencing was performed on one lane of Illumina NovaSeq S1 flowcell. We obtained a mean (±SD) of 42M (±5) reads per sample.

2.15. Longitudinal differential expression across adipogenesis

We analyzed the longitudinal trajectory patterns of 124 known pathway genes involved in adipogenesis (https://www.wikipathways.org/pathways/WP236.html) and expressed in these adipogenesis data, as well as 5 additional demethylase and methylase genes (*UHRF1*, *TET1*, *TET2*, *TET3*, and *TDG*) also expressed in these data using ImpulseDE2 v0.99.10[45] across the 6 adipogenesis time points. We used the runImpulseDE2 function with parameters

boolCaseCtrl=FALSE, *boolIdentifyTransients*=TRUE, and *scaNProc*=1 on the respective RNA-seq gene expression counts. All P values were corrected for multiple testing using FDR<0.05.

2.16. Longitudinal trajectories of co-expressed genes

To search for longitudinal co-expression patterns among the key demethylase and methylase genes across human adipogenesis, we ran DPGP v0.1[25] to cluster genes by their expression trajectories. We only included the genes that were identified as significantly longitudinally DE (FDR<0.05) during human adipogenesis from ImpulseDE2[45], as described above.

2.17. Overrepresentation of GWAS variants in DMRs and compartments

To examine the overrepresentation of GWAS variants for WHRadjBMI, BMI, MASLD, and CRP in cell-type level DMRs and compartments, we selected independent GWAS variants by LD-clumping the GWAS significant variants ($P<5\times10^{-8}$) to an $R^2<0.2$ per a 250-kb window. We then compared the proportion of independent GWAS variants landing in each epigenomic region of interest against the proportion of the genome occupied by that region, using one-tailed hypergeometric test. Significant enrichment was defined as hypergeometric $P<0.05$.

2.18. Transcriptomic impact of GWAS variants in cell-type DMRs

We investigated what genes are associated with the WHRadjBMI GWAS SNPs residing in the hypo- and hyper-methylated regions in each SAT cell-type by overlapping these GWAS variants with bulk SAT *cis*-eQTL variants detected in the GTEx v10 cohort[46]. We then assessed and cataloged these *cis*-eQTL target genes (eGenes), regulated by the overlapping variants, based on their genetic relevance to abdominal obesity (using WHRadjBMI as a well-established proxy)

employing the HuGE scoring[26]. HuGE scores indicate the strength of the genetic evidence per gene for its contribution to the cardiometabolic trait[26]. For each set of cell-type level DMRs, we conducted 10,000 permutations to examine whether the percentage of above-mentioned eGenes around the DMRs, showing "very strong" or higher genetic evidence for WHRadjBMI (HuGE scores≥30)[26], was significantly higher than the null distributions. For each set of genes, significance was determined by $P_{perm10,000}<0.05$.

Supplementary Figures

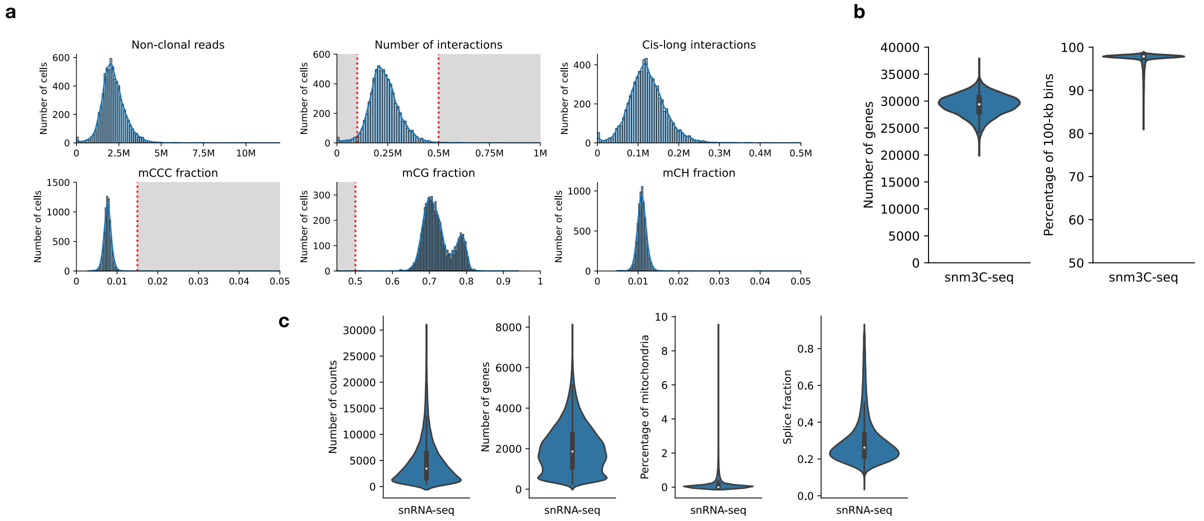

Supplementary Figure 1. Distribution of the QC metrics used in preprocessing SAT snm3C-seq and snRNA-seq nuclei. a, Distribution of the QC metrics used in filtering the snm3C-seq nuclei (n=6,910) with the shaded areas and red dashed lines indicating filtering cutoffs. The snm3C-seq nuclei that passed QC (n=6,652) have a mean (±SD) of 2,215,681 (±65,644) non-clonal reads, 236,849 (±65,220) total interactions, 124,911 (±40,280) *cis*-long interactions (i.e., intra-chromosomal interactions >10-kb), 0.0077% (±0.0008) mCCC non-conversion rate, 0.724% (±0.038) mCG rate, and 0.011% (±0.001) mCH rate. **b,** Violin plots showing the entire distributions of genomic coverage features for the snm3C-seq nuclei that passed QC (n=6,652), with a mean (±SD) coverage of 29,251 (±1,953) genes and 97.3% (±1.7) non-overlapping 100-kb bins. **c,** Violin plots showing the entire distributions of transcriptomic features for the snRNA-seq nuclei that passed the QC (n=29,423), with a mean (±SD) of 4,741 (±4,334) counts, 2,001 (±1,154) number of expressed genes, 0.2% (±0.6) mitochondria expression, and 29.9% (±12.9) splice fraction. The center of the box inside the violin plot represents the median; the bounds of the box indicate the 25% and 75% percentiles, while the whiskers show the minimum and maximum values within 1.5 times the interquartile range. QC, quality control; and SAT, subcutaneous adipose tissue.

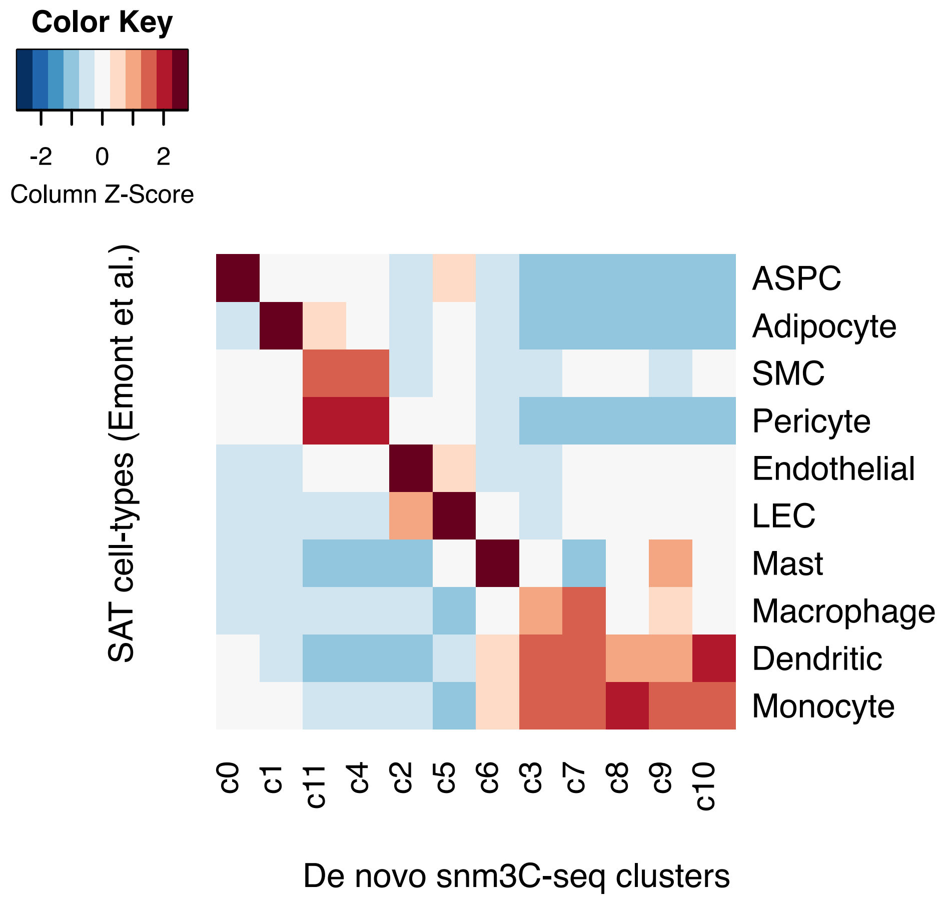

Supplementary Figure 2. *De novo* snm3C-seq cluster annotations with external SAT reference markers. Pairwise overlap fractions between the top (up to 50) *de novo* snm3C-seq cluster marker genes (on the x-axis) and those of relevant cell-types from Emont et al.[3] (y-axis). SAT, subcutaneous adipose tissue.

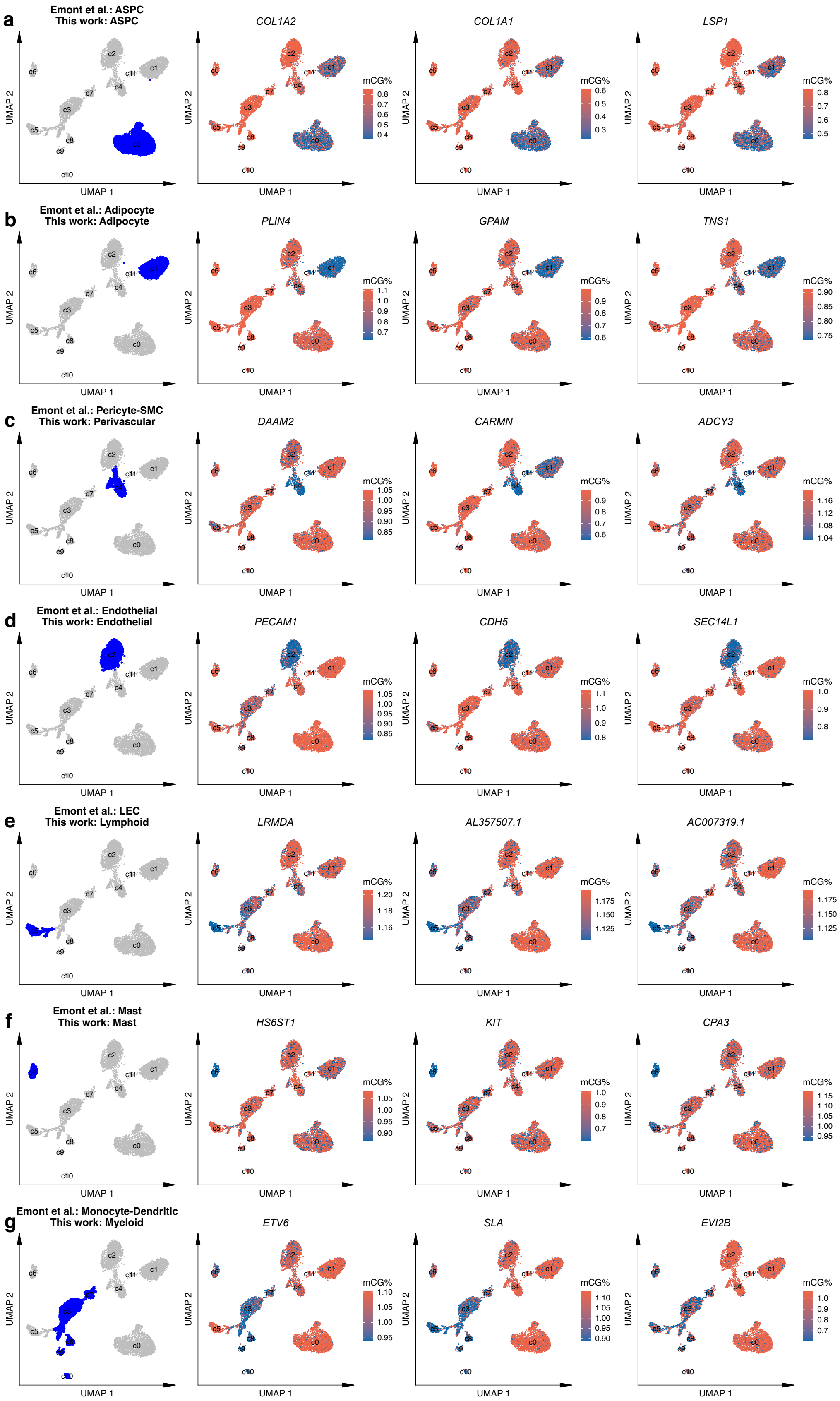

a
Emont et al.: ASPC
This work: ASPC
COL1A2
COL1A1
LSP1
mCG%
0.8
0.7
0.6
0.5
0.4
0.6
0.5
0.4
0.3
0.8
0.7
0.6
0.5
b
Emont et al.: Adipocyte
This work: Adipocyte
PLIN4
GPAM
TNS1
1.1
1.0
0.9
0.8
0.7
0.9
0.8
0.7
0.6
0.90
0.85
0.80
0.75
c
Emont et al.: Pericyte-SMC
This work: Perivascular
DAAM2
CARMN
ADCY3
1.05
1.00
0.95
0.90
0.85
0.9
0.8
0.7
0.6
1.16
1.12
1.08
1.04
d
Emont et al.: Endothelial
This work: Endothelial
PECAM1
CDH5
SEC14L1
1.05
1.00
0.95
0.90
0.85
1.1
1.0
0.9
0.8
1.0
0.9
0.8
e
Emont et al.: LEC
This work: Lymphoid
LRMDA
AL357507.1
AC007319.1
1.20
1.18
1.16
1.175
1.150
1.125
1.175
1.150
1.125
f
Emont et al.: Mast
This work: Mast
HS6ST1
KIT
CPA3
1.05
1.00
0.95
0.90
1.0
0.9
0.8
0.7
1.15
1.10
1.05
1.00
0.95
g
Emont et al.: Monocyte-Dendritic
This work: Myeloid
ETV6
SLA
EVI2B
1.10
1.05
1.00
0.95
1.10
1.05
1.00
0.95
0.90
1.0
0.9
0.8
0.7
UMAP 1
UMAP 2
c0
c1
c2
c3
c4
c5
c6
c7
c8
c9
c10
c11

Supplementary Figure 3. Gene body mCG profiles of marker genes shared between the *de novo* snm3C-seq clusters and external SAT reference cell-types. a-g, Uniform manifold approximation and projection (UMAP) visualization highlighting the cluster(s) being annotated, with corresponding cell-type annotations from Emont et al.[3] (left) and the gene body mCG ratio, normalized per cell (right) for ASPC marker genes *COL1A2*, *COL1A1*, and *LSP1* (a); adipocyte marker genes *PLIN4*, *GPAM*, and *TNS1* (b); perivascular cell marker genes *DAAM2*, *CARMN*, and *ADCY3* (c); endothelial cell marker genes *PECAM1*, *CDH5*, and *SEC14L1* (d); lymphoid cell marker genes *LRMDA*, *AL357507.1*, and *AC007319.1* (e); mast cell marker genes *HS6ST1*, *KIT*, and *CPA3* (f); and myeloid cell marker genes *ETV6*, *SLA*, and *EVI2B* (g). SAT, subcutaneous adipose tissue; and ASPC, adipose stem and progenitor cell.

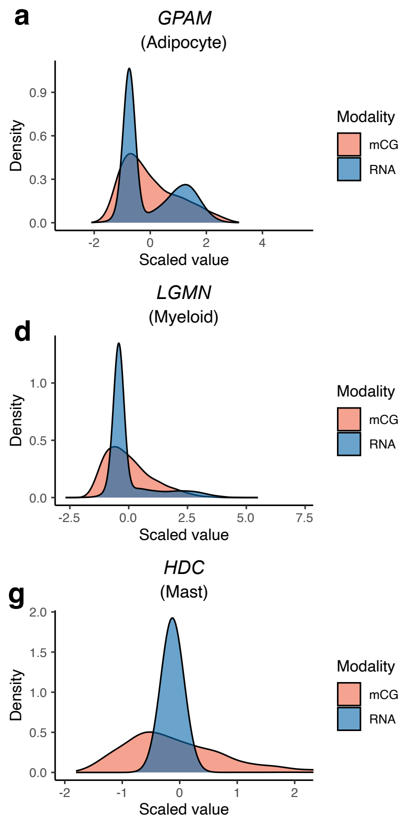

Supplementary Figure 4. Comparing the distributions of the standardized mCG fractions and expression values across cells profiled by snm3C-seq and snRNA-seq. a-g, Density plots showing the mCG fractions and expression values after standardization, superimposed on each other, for a selection of marker genes included in the CCA framework to align the modalities. (a) Adipocyte marker gene *GPAM*. (b) Perivascular marker gene *NOTCH3*. (c) ASPC marker gene *COL5A1*. (d) Myeloid cell marker gene *LGMN*. (e) Endothelial marker gene *CDH5*. (f) Lymphoid cell marker gene *CD247*. (g) Mast cell marker gene *HDC*. ASPC, adipose stem and progenitor cell; and CCA, canonical correlation analysis.

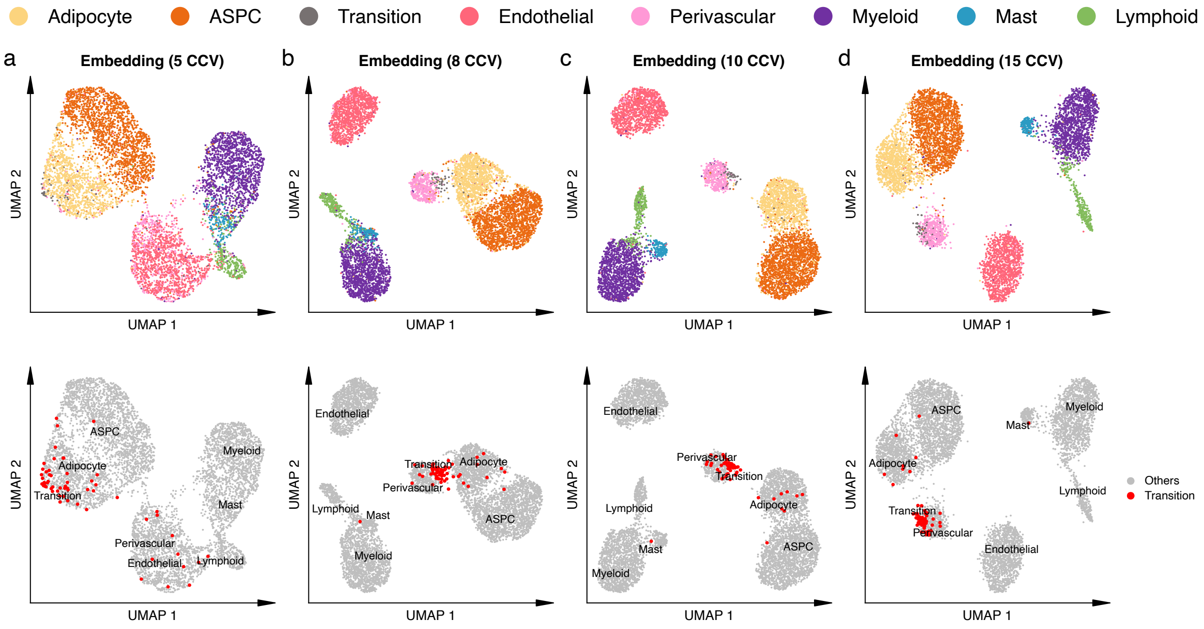

Supplementary Figure 5. Visualization of the top CCVs embeddings and dynamics of the cluster membership of the transitional cells. a-d, Uniform manifold approximation and projection (UMAP) visualizations of the embeddings for snm3C-seq and snRNA-seq cells, constructed using various top k CCVs: (a) k=5, (b) k=8, (c) k=10, and (d) k=15. Cells are colored by the SAT cell-type annotation from Figure 2d (left) and by their membership in the transition cell-type cluster (right). CCV, canonical correlation vector; SAT, subcutaneous adipose tissue; and ASPC, adipose stem and progenitor cell.

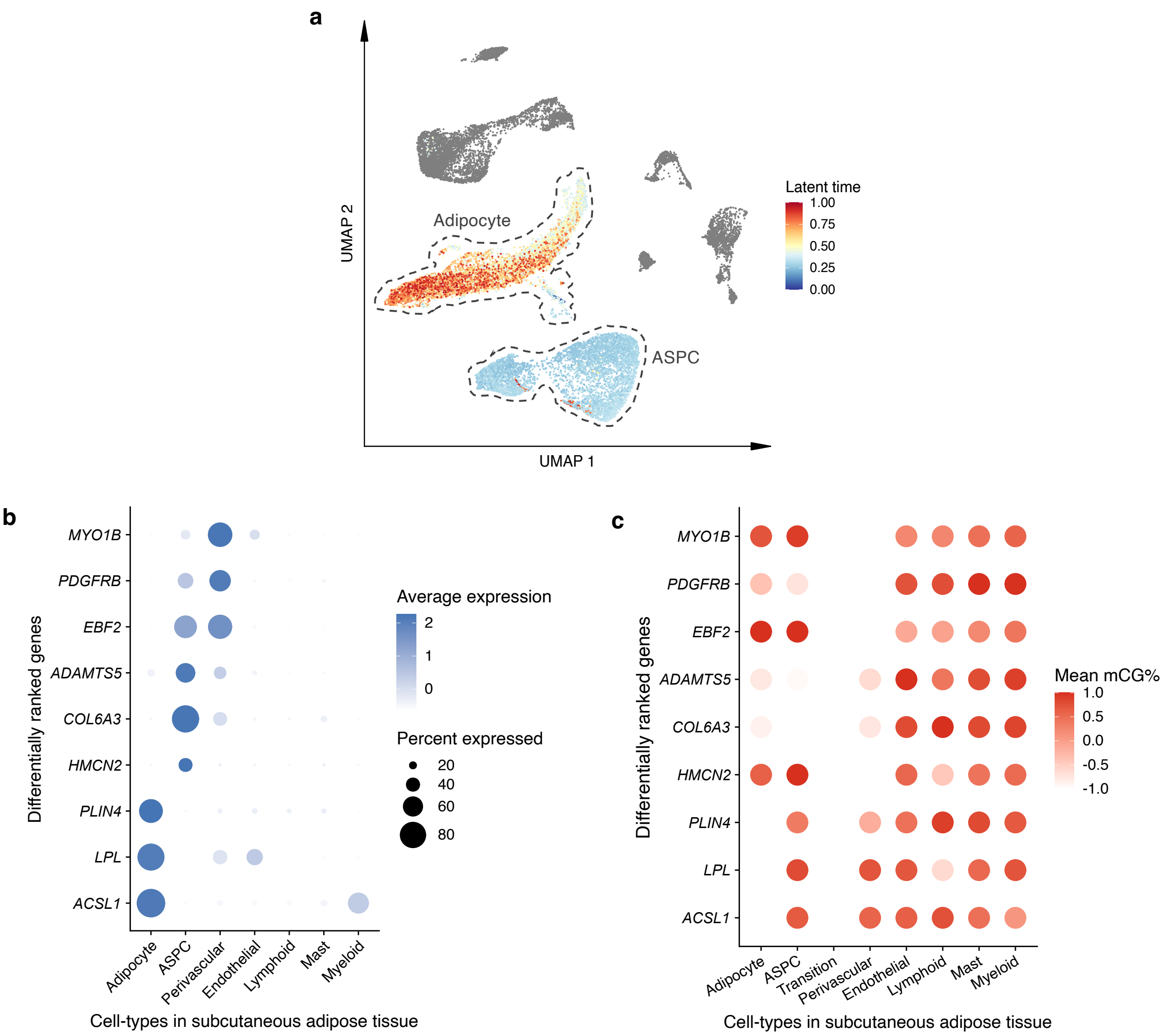

Supplementary Figure 6. Latent time analysis and visualization of the expression and methylation profiles of representative genes involved in the dynamic time trajectories. a, Uniform manifold approximation and projection (UMAP) visualizations of the embeddings for snRNA-seq cells, colored by the estimated latent time on cells annotated as ASPCs and adipocytes (highlighted by the dashed lines). **b-c,** Dot plots depicting the gene expression (b) and gene body mCG profiles (c) for select differentially ranked genes, which are also marker genes for the transition cell-type, stratified by the SAT cell-types. Dot color represents the average log-transformed counts per million normalized gene expression (left) and the average gene body mCG

ratio, normalized per cell (right). ASPC, adipose stem and progenitor cell; and SAT, subcutaneous adipose tissue.

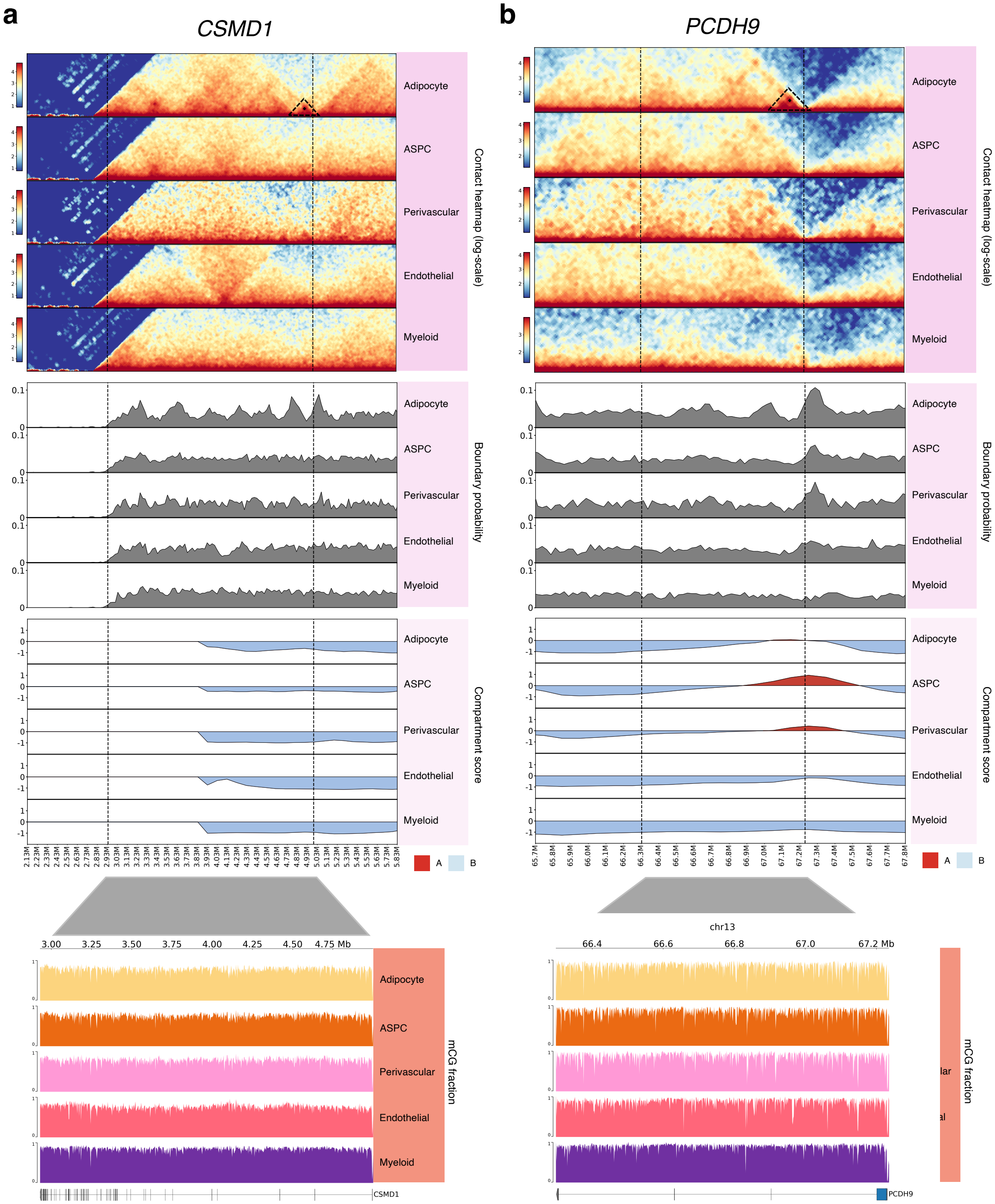

Supplementary Figure 7. Browser-based stacked views of multimodal data for adipocyte marker genes in the five major SAT cell-types. a-b, Similar to Extended Data Figure 8 except on the *CSMD1* (a) and *PCDH9* (b). The black dashed triangles in the contact heatmap highlight

differential loop domains near the transcription start sites that are present only in adipocytes. SAT, subcutaneous adipose tissue; and ASPC, adipose stem and progenitor cell.

References

1. Tian, W. *et al.* Single-cell DNA methylation and 3D genome architecture in the human brain. *Science* **382**, eadf5357 (2023).
2. Lee, D.-S. *et al.* Simultaneous profiling of 3D genome structure and DNA methylation in single human cells. *Nat. Methods* **16**, 999–1006 (2019).
3. Emont, M. P. *et al.* A single-cell atlas of human and mouse white adipose tissue. *Nature* **603**, 926–933 (2022).
4. Nothjunge, S. *et al.* DNA methylation signatures follow preformed chromatin compartments in cardiac myocytes. *Nat. Commun.* **8**, 1667 (2017).
5. Angueira, A. R. *et al.* Defining the lineage of thermogenic perivascular adipose tissue. *Nat. Metab.* **3**, 469–484 (2021).
6. Takashima, Y. *et al.* PDGF receptor signal mediates the contribution of Nestin-positive cell lineage to subcutaneous fat development. *Biochem. Biophys. Res. Commun.* **658**, 27–35 (2023).
7. Oguri, Y. *et al.* CD81 Controls Beige Fat Progenitor Cell Growth and Energy Balance via FAK Signaling. *Cell* **182**, 563-577.e20 (2020).
8. Wang, W. *et al.* Ebf2 is a selective marker of brown and beige adipogenic precursor cells. *Proc. Natl Acad. Sci. USA* **111**, 14466–71 (2014).
9. Angueira, A. R. *et al.* Early B Cell Factor Activity Controls Developmental and Adaptive Thermogenic Gene Programming in Adipocytes. *Cell Rep.* **30**, 2869-2878.e4 (2020).
10. Lambert, S. A. *et al.* The Human Transcription Factors. *Cell* **172**, 650–665 (2018).
11. Metzger, D. *et al.* Functional role of RXRs and PPARgamma in mature adipocytes. *Prostaglandins Leukot. Essent. Fatty Acids* **73**, 51–8 (2005).

12. Heinz, S. *et al.* Simple combinations of lineage-determining transcription factors prime cis-regulatory elements required for macrophage and B cell identities. *Mol. Cell* **38**, 576–89 (2010).

13. Dutta, A. B. *et al.* Kinetic networks identify TWIST2 as a key regulatory node in adipogenesis. *Genome Res.* **33**, 314–331 (2023).

14. Kumari, R. *et al.* SMAD2 and SMAD3 differentially regulate adiposity and the growth of subcutaneous white adipose tissue. *FASEB J.* **35**, e22018 (2021).

15. Zaragosi, L.-E. *et al.* Activin a plays a critical role in proliferation and differentiation of human adipose progenitors. *Diabetes* **59**, 2513–21 (2010).

16. Gombart, A. F., Grewal, J. & Koeffler, H. P. ATF4 differentially regulates transcriptional activation of myeloid-specific genes by C/EBPepsilon and C/EBPalpha. *J. Leukoc. Biol.* **81**, 1535–47 (2007).

17. Jääskeläinen, I. *et al.* Collagens Regulating Adipose Tissue Formation and Functions. *Biomedicines* **11**, (2023).

18. Wang, N., Zhang, J., Lv, H. & Liu, Z. Regulation of COL1A2, AKT3 genes, and related signaling pathway in the pathology of congenital talipes equinovarus. *Front. Pediatr.* **10**, 890109 (2022).

19. Menezes, M. J. *et al.* The extracellular matrix protein laminin α2 regulates the maturation and function of the blood-brain barrier. *J. Neurosci.* **34**, 15260–80 (2014).

20. Lluch, A. *et al.* A novel long non-coding RNA connects obesity to impaired adipocyte function. *Mol. Metab.* **90**, 102040 (2024).

21. Kar, A. *et al.* Age-dependent genes in adipose stem and precursor cells affect regulation of fat cell differentiation and link aging to obesity via cellular and genetic interactions. *Genome Med.* **16**, 19 (2024).

22. Pan, D. Z. *et al.* Identification of TBX15 as an adipose master trans regulator of abdominal obesity genes. *Genome Med.* **13**, 123 (2021).

23. Laakso, M. *et al.* The Metabolic Syndrome in Men study: a resource for studies of metabolic and cardiovascular diseases. *J. Lipid Res.* **58**, 481–493 (2017).

24. Sun, S., Ji, Y., Kersten, S. & Qi, L. Mechanisms of inflammatory responses in obese adipose tissue. *Annu. Rev. Nutr.* **32**, 261–86 (2012).

25. McDowell, I. C. *et al.* Clustering gene expression time series data using an infinite Gaussian process mixture model. *PLoS Comput. Biol.* **14**, e1005896 (2018).

26. Dornbos, P. *et al.* Evaluating human genetic support for hypothesized metabolic disease genes. *Cell Metab.* **34**, 661–666 (2022).

27. Frankish, A. *et al.* GENCODE 2021. *Nucleic Acids Res.* **49**, D916–D923 (2021).

28. Dobin, A. *et al.* STAR: ultrafast universal RNA-seq aligner. *Bioinformatics* **29**, 15–21 (2013).

29. Alvarez, M. *et al.* Enhancing droplet-based single-nucleus RNA-seq resolution using the semi-supervised machine learning classifier DIEM. *Sci. Rep.* **10**, 11019 (2020).

30. Deal, M. *et al.* An abdominal obesity missense variant in the adipocyte thermogenesis gene TBX15 is implicated in adaptation to cold in Finns. *Am. J. Hum. Genet.* **111**, 2542–2560 (2024).

31. Hao, Y. *et al.* Integrated analysis of multimodal single-cell data. *Cell* **184**, 3573-3587.e29 (2021).

32. Yang, S. *et al.* Decontamination of ambient RNA in single-cell RNA-seq with DecontX. *Genome Biol.* **21**, 57 (2020).

33. Kang, H. M. *et al.* Multiplexed droplet single-cell RNA-sequencing using natural genetic variation. *Nat. Biotechnol.* **36**, 89–94 (2018).

34. McGinnis, C. S., Murrow, L. M. & Gartner, Z. J. DoubletFinder: Doublet Detection in Single-Cell RNA Sequencing Data Using Artificial Nearest Neighbors. *Cell Syst.* **8**, 329-337.e4 (2019).

35. Liu, H. *et al.* Single-cell DNA methylome and 3D multi-omic atlas of the adult mouse brain. *Nature* **624**, 366–377 (2023).

36. Amemiya, H. M., Kundaje, A. & Boyle, A. P. The ENCODE Blacklist: Identification of Problematic Regions of the Genome. *Sci. Rep.* **9**, 9354 (2019).

37. Liu, H. *et al.* DNA methylation atlas of the mouse brain at single-cell resolution. *Nature* **598**, 120–128 (2021).

38. Korsunsky, I. *et al.* Fast, sensitive and accurate integration of single-cell data with Harmony. *Nat. Methods* **16**, 1289–1296 (2019).

39. Bergen, V., Lange, M., Peidli, S., Wolf, F. A. & Theis, F. J. Generalizing RNA velocity to transient cell states through dynamical modeling. *Nat. Biotechnol.* **38**, 1408–1414 (2020).

40. ENCODE Project Consortium. An integrated encyclopedia of DNA elements in the human genome. *Nature* **489**, 57–74 (2012).

41. Zhang, J. *et al.* An integrative ENCODE resource for cancer genomics. *Nat. Commun.* **11**, 3696 (2020).

42. Moore, J. E., Pratt, H. E., Purcaro, M. J. & Weng, Z. A curated benchmark of enhancer-gene interactions for evaluating enhancer-target gene prediction methods. *Genome Biol.* **21**, 17 (2020).

43. Zhang, J. *et al.* DiNeR: a Differential graphical model for analysis of co-regulation Network Rewiring. *BMC Bioinformatics* **21**, 281 (2020).

44. Law, C. W., Chen, Y., Shi, W. & Smyth, G. K. voom: Precision weights unlock linear model analysis tools for RNA-seq read counts. *Genome Biol.* **15**, R29 (2014).

45. Fischer, D. S., Theis, F. J. & Yosef, N. Impulse model-based differential expression analysis of time course sequencing data. *Nucleic Acids Res.* **46**, e119 (2018).

46. GTEx Consortium. The GTEx Consortium atlas of genetic regulatory effects across human tissues. *Science* **369**, 1318–1330 (2020).
