# Supplementary material for: Single-cell DNA methylome and 3D genome atlas of human subcutaneous adipose tissue
Source: Nat Genet. 2025 Aug 20;57(9):2238–49. doi: 10.1038/s41588-025-02300-4 (PMC12373012; doi:10.1038/s41588-025-02300-4)
Supplement: Supplementary file 2 — Reporting Summary [file 41588_2025_2300_MOESM2_ESM.pdf]

nature portfolio

Corresponding author(s): Päivi Pajukanta
Last updated by author(s): Jun 9, 2025

Reporting Summary

Nature Portfolio wishes to improve the reproducibility of the work that we publish. This form provides structure for consistency and transparency in reporting. For further information on Nature Portfolio policies, see our Editorial Policies and the Editorial Policy Checklist.

Statistics

For all statistical analyses, confirm that the following items are present in the figure legend, table legend, main text, or Methods section.

n/a	Confirmed	
☐	☒	The exact sample size (*n*) for each experimental group/condition, given as a discrete number and unit of measurement
☐	☒	A statement on whether measurements were taken from distinct samples or whether the same sample was measured repeatedly
☐	☒	The statistical test(s) used AND whether they are one- or two-sided<br>*Only common tests should be described solely by name; describe more complex techniques in the Methods section.*
☐	☒	A description of all covariates tested
☐	☒	A description of any assumptions or corrections, such as tests of normality and adjustment for multiple comparisons
☐	☒	A full description of the statistical parameters including central tendency (e.g. means) or other basic estimates (e.g. regression coefficient) AND variation (e.g. standard deviation) or associated estimates of uncertainty (e.g. confidence intervals)
☐	☒	For null hypothesis testing, the test statistic (e.g. *F*, *t*, *r*) with confidence intervals, effect sizes, degrees of freedom and *P* value noted<br>*Give P values as exact values whenever suitable.*
☒	☐	For Bayesian analysis, information on the choice of priors and Markov chain Monte Carlo settings
☐	☒	For hierarchical and complex designs, identification of the appropriate level for tests and full reporting of outcomes
☐	☒	Estimates of effect sizes (e.g. Cohen's *d*, Pearson's *r*), indicating how they were calculated

Our web collection on statistics for biologists contains articles on many of the points above.

Software and code

Policy information about availability of computer code

Data collection	No software was used to collect the data.
Data analysis	All packages and software used in this study were from their publicly available sources, as outlined in the Methods. No custom code was used.<br><br>As described in the Methods, quality control of genotype data (Tilkka participants) was done using PLINK v1.9. We used the HRC reference panel version r1.1 2016 to perform genotype imputation against the Michigan imputation server. Eagle v2.4 was used for haplotype phasing, and minimac4 for genotype imputation. We aligned the raw SAT snRNA-seq data against GRCh38 human reference genome with STAR v2.7.10b. We evaluated the quality of the mapped data using FastQC v0.11.9. We used DIEM v2.4.0 to remove empty droplets as well as nuclei. We used DecontX from the celda R package v1.14.2 to remove reads from ambient RNA. We used demuxlet v2 from popscle software tool to identify the originating individuals. We employed DoubletFinder v2.0.3 to remove doublets. We used Seurat v4.3.0 to normalize gene counts, identify variable genes, perform PCA analysis, and cluster the nuclei. We annotated the clusters with SingleR v1.8.1.<br><br>We used WebGestalt 2024 for pathway enrichment analysis. We analyzed longitudinal trajectory patterns of genes and clustered genes by their expression trajectories with ImpulseDE2 v0.99.10 and DPGP v0.1, respectively. We used scVelo v0.3.2 and GeneOverlap v1.36.0 for the adipogenesis trajectory analysis. We employed edgeR v3.40.2 and limma-voom 3.54.2 for the SAT bulk differential expression analysis in the METSIM cohort.<br><br>Sequencing reads of snm3C-seq were mapped using Taurus-MH (https://github.com/luogenomics/Taurus-MH). We used ALLCools package v.1.0.23 for the majority of the analyses related to the DNA methylome data modality and scHicluster v.1.3.5 for those on chromosome contact data. We applied Harmony v.0.0.9 to remove sample-level batch effects. We used the MethylPy package implemented in the ALLCools package v.1.0.23 to detect differentially methylated sites and regions. We used the CCA framework implemented in Seurat v.4.1.0 to align

snRNA-seq and snm3C-seq nuclei. We used HOMER v4.11.1 to predict transcription factor binding motif enrichment analysis and the circlize package v0.4.16 to visualize the results. We used dcHic v2.1 for cell-type level differential compartment analysis and TopDom v0.0.2 to detect domain boundaries in each nucleus.

Finally, we performed LD-clumping using PLINK v1.9 to construct independent marker sets and BOLT-LMM v2.3.6 to calculate GWAS summary statistics. For additional details, please see the Methods.

For manuscripts utilizing custom algorithms or software that are central to the research but not yet described in published literature, software must be made available to editors and reviewers. We strongly encourage code deposition in a community repository (e.g. GitHub). See the Nature Portfolio guidelines for submitting code & software for further information.

Data

Policy information about availability of data

All manuscripts must include a data availability statement. This statement should provide the following information, where applicable:

- Accession codes, unique identifiers, or web links for publicly available datasets
- A description of any restrictions on data availability
- For clinical datasets or third party data, please ensure that the statement adheres to our policy

The data that support the findings in this manuscript are available from the UK Biobank. However, restrictions apply to the availability of these data, which were used in this study under UK Biobank Application number 33934. UK Biobank data are available for bona fide researchers through the application process: https://www.ukbiobank.ac.uk/learn-more-about-uk-biobank/contact-us. The snm3C-seq and snRNA-seq data from the Tilkka cohort are available in the NIH Gene Expression Omnibus (GEO), under accession number GSE297267 (https://www.ncbi.nlm.nih.gov/geo/query/acc.cgi?acc=GSE297267), along with the epigenomic annotations of SAT DMRs, compartments, domains, and loops, characterized in this study. GRCh38 reference genome is available through the UCSC genome browser (https://hgdownload.soe.ucsc.edu/goldenPath/hg38/bigZips/). The bulk RNA-seq data from the primary human preadipocyte differentiation experiment was previously made available in GEO, under accession number GSE249195 (https://www.ncbi.nlm.nih.gov/geo/query/acc.cgi?acc=GSE249195). The METSIM SAT bulk RNA-seq data52 are available in GEO, under accession number GSE135134 (https://www.ncbi.nlm.nih.gov/geo/query/acc.cgi?acc=GSE135134). Bulk SAT cis-eQTL variants are available from the GTEx v10 cohort. ChIP-seq datasets are publicly available on the ENCODE portal with the following accession numbers: ENCSR000BGY (https://www.encodeproject.org/experiments/ENCSR000BGY/), ENCSR177VFS (https://www.encodeproject.org/experiments/ENCSR177VFS/), and ENCSR490LWA (https://www.encodeproject.org/experiments/ENCSR490LWA/). Source data files and GWAS summary statistics from this study are available on Zenodo (https://zenodo.org/records/15318595).

Research involving human participants, their data, or biological material

Policy information about studies with human participants or human data. See also policy information about sex, gender (identity/presentation), and sexual orientation and race, ethnicity and racism.

Reporting on sex and gender	Self reported sex information was available for all individuals in the Tilkka and METSIM cohorts. In UKB, we also used the self reported sex information. For the PRS analyses, we included sex as a covariate for the analyses with all individuals. Additional details are available in the Methods.<br><br>Tilkka cohort is comprised of 8 females. The UKB cohorts includes 54.0% female. The METSIM only includes unrelated men.
Reporting on race, ethnicity, or other socially relevant groupings	All study participants are individuals of European ancestry. No socially relevant groupings were considered.
Population characteristics	Eight Finnish females without obesity underwent abdominal liposuction at Tilkka Hospital, Helsinki, Finland. We performed single nucleus RNA-seq on 8 subcutaneous adipose tissue (SAT) biopsies and snm3C-seq on 5 SAT biopsies taken at the liposuction. The mean age for the snRNA-seq cohort is 44.5 years (SD=6.03 years), and for the snm3C-seq cohort, 44.4 years (SD=4.78 years). Age was not used as a covariate in any of the analyses of the Tilkka cohort. The UK Biobank consists of ~500,000 individuals with genotypes and phenotypes and includes a broad range of ages. In this study, we only included the unrelated European-origin UKB participants in our analysis (n=391,701; 54% females; mean age=56.9 years, SD=8.0). The METSIM bulk RNA-seq data comprise 335 Finnish unrelated men (mean age=54.1 years, SD=4.9). Additional details are available in Methods.
Recruitment	The participants in Tilkka study were recruited at Tilkka Hospital, Helsinki, Finland. The UK Biobank study recruited individuals of various ages and backgrounds across 22 assessment centers. The participants of the METSIM SAT bulk RNA-seq cohort were recruited at the Kuopio University Hospital and University of Eastern Finland, Kuopio, Finland.
Ethics oversight	Tilkka cohort<br>The Tilkka study was approved by the Helsinki University Hospital Ethics Committee (approval no. HUS/1039/2019) and all participants provided a written informed consent. All research conformed to the principles of the Declaration of Helsinki. No compensation was provided to the participants.<br><br>UK Biobank cohort<br>The UKB study was approved by the North West Multi-centre Research Ethics Committee (approval no. 21/NW/0157). All participants gave written informed consent. All research conformed to the principles of the Declaration of Helsinki. No compensation was provided to the participants.<br><br>METSIM cohort<br>All METSIM men provided written informed consent, and the METSIM study was approved by the Ethics Committee of the Northern Savo Hospital District (approval no. 171/2004). All research conformed to the principles of the Declaration of

Helsinki. No compensation was provided to the participants.

Note that full information on the approval of the study protocol must also be provided in the manuscript.

Field-specific reporting

Please select the one below that is the best fit for your research. If you are not sure, read the appropriate sections before making your selection.

☒ Life sciences ☐ Behavioural & social sciences ☐ Ecological, evolutionary & environmental sciences

For a reference copy of the document with all sections, see nature.com/documents/nr-reporting-summary-flat.pdf

Life sciences study design

All studies must disclose on these points even when the disclosure is negative.

Sample size	No statistical method was used to predetermine the sample size of the snm3C-seq cohort; however, our sample sizes and number of cells profiled are similar to those reported in previous snm3C-seq publications (Lee et al. Nat Methods. 2019;16:999-1006). We applied snm3C-seq on SAT biopsies from 5 participants and snRNA-seq on SAT biopsies from 8 participants. For the polygenic risk score analyses, we used genotype and phenotype data from the 391,701 unrelated individuals of European-origin of the UK Biobank cohort. For the METSIM SAT bulk RNA-seq analyses, all available RNA-seq samples were used (n=335).
Data exclusions	No samples were excluded from the Tilkka cohort. In the UKB and METSIM cohorts, related individuals were excluded to avoid confounding due to relatedness.
Replication	Results obtained from SAT snm3C-seq and snRNA-seq were not replicated due to the unique nature of these datasets. Results reported from the UKB GWAS analyses were not replicated due to the unprecedentedly large sample size of the cohort. Correlations observed with the METSIM bulk RNA-seq data were not replicated due to the unique refined phenotype profiles of the METSIM cohort. Additional details are available in Methods.
Randomization	N/A. This is an observational study, so no randomization was performed.
Blinding	Data collection and analysis were performed blind to the conditions of the experiments.

Reporting for specific materials, systems and methods

We require information from authors about some types of materials, experimental systems and methods used in many studies. Here, indicate whether each material, system or method listed is relevant to your study. If you are not sure if a list item applies to your research, read the appropriate section before selecting a response.

Materials & experimental systems

n/a	Involved in the study	
☒	☐	Antibodies
☒	☐	Eukaryotic cell lines
☒	☐	Palaeontology and archaeology
☒	☐	Animals and other organisms
☒	☐	Clinical data
☒	☐	Dual use research of concern
☒	☐	Plants

Methods

n/a	Involved in the study	
☒	☐	ChIP-seq
☒	☐	Flow cytometry
☒	☐	MRI-based neuroimaging

Plants

Seed stocks	*Report on the source of all seed stocks or other plant material used. If applicable, state the seed stock centre and catalogue number. If plant specimens were collected from the field, describe the collection location, date and sampling procedures.*
Novel plant genotypes	*Describe the methods by which all novel plant genotypes were produced. This includes those generated by transgenic approaches, gene editing, chemical/radiation-based mutagenesis and hybridization. For transgenic lines, describe the transformation method, the number of independent lines analyzed and the generation upon which experiments were performed. For gene-edited lines, describe the editor used, the endogenous sequence targeted for editing, the targeting guide RNA sequence (if applicable) and how the editor was applied.*
Authentication	*Describe any authentication procedures for each seed stock used or novel genotype generated. Describe any experiments used to assess the effect of a mutation and, where applicable, how potential secondary effects (e.g. second site T-DNA insertions, mosiacism, off-target gene editing) were examined.*
